# Small-Target Ship Detection with Joint Spatio-Temporal Features Across Multiple Frames

**DOI:** 10.3390/s26113588

**Published:** 2026-06-04

**Authors:** Ye Qian, Zhen Hu, Bo Zhang, Wenguang Yang, Qian Chen

**Affiliations:** 1 Taihu Laboratory of Deepsea Technological Science, Wuxi 214000, China; huzhen702@163.com (Z.H.); xbeagle702@126.com (B.Z.); 2 China Ship Scientific Research Center, Wuxi 214000, China; 3 State Key Laboratory of Deep-Sea Manned Vehicles, Wuxi 214000, China; 4 Nanjing Research Institute of Electronic Engineering, Nanjing 210000, China; ywgsyudent@163.com; 5 School of Electronic and Optical Engineering, Nanjing University of Science and Technology, Nanjing 210094, China; chenq@njust.edu.cn; 6 School of Information and Communication Engineering, North University of China, Taiyuan 030051, China

**Keywords:** small-target ship detection, sea–sky background suppression, multi-scale feature enhancement, temporal trajectory analysis

## Abstract

Detecting small ship targets in sea–sky background environments is challenging due to interference from clouds, islands, sea clutter, and the limited spatial information in long-range infrared imagery. To address these issues, this paper proposes a robust detection framework that integrates multi-scale spatial feature enhancement with temporal trajectory analysis. First, a candidate target extraction method based on a multi-scale differential histogram of oriented gradients is introduced. By exploiting gradient distribution differences between targets and surrounding backgrounds, our method effectively enhances target responses while suppressing structured background edges. This response is further fused with a log-spectrum-based saliency map to improve target contrast and reduce clutter. Next, a candidate trajectory extraction algorithm based on inverse optical flow matching is developed to utilize temporal consistency. Optical flow-based grayscale compensation predicts target intensity changes between frames, while Kalman filtering estimates motion states and performs trajectory association. Finally, a multi-feature trajectory filtering strategy is designed, combining motion entropy stability, peak signal-to-noise ratio, and trajectory lifecycle to distinguish true targets from false alarms. Experimental results on eight infrared maritime sequences demonstrate superior performance. The proposed method achieves an average Background Suppression Factor (BSF) of 45.2 and an average Signal-to-Clutter Ratio Gain (SCRG) of 22.3 × 10^3^, representing a substantial improvement over all baseline algorithms. Receiver Operating Characteristic analysis further confirms a mean detection rate exceeding 90% at a false-alarm rate of 10^−3^ across all sequences, confirming improved detection performance and robustness in complex maritime environments.

## 1. Introduction

Ship target detection plays a crucial role in applications such as maritime traffic surveillance and naval situational awareness [1]. However, under conditions of low ship velocity, poor imaging quality, complex sea states, or limited observation angles, ship wakes are often indistinct or entirely absent. In such scenarios, detection must rely solely on the structural characteristics of the ship hull. According to the definition proposed by the Society of Photo-Optical Instrumentation Engineers (SPIE) in 1989 [2], a target is classified as a small target if its contrast is below 15%, its signal-to-noise ratio (SNR) is less than 1.5, and it occupies less than 0.15% of the total image area. Consequently, ship detection under long-range imaging conditions can be regarded as a typical small-target detection problem. Existing ship-borne small-target detection methods face significant challenges, including target sparsity, low contrast, and strong environmental interference [3]. These methods can generally be categorized into two groups: single-frame methods and multi-frame methods.

Single-frame small-target detection aims to identify targets within an individual image. It is particularly suitable for static scenes or scenarios where temporal information is unavailable, offering advantages such as high computational efficiency and broad applicability in practical systems. Existing single-frame methods can generally be categorized into four groups: background estimation-based methods, visual attention-based methods, sparse and low-rank representation methods, and deep learning-based methods.

Background estimation-based methods utilize prior knowledge of background consistency to model and estimate the background, and then enhance the target by subtracting the estimated background from the original image. Representative approaches include the Top-Hat filter [4], LMS filter [5], and max-mean/max-median filters [6]. More recently, Li et al. [7] proposed a tensor-based least mean square (TLMS) method, Fan et al. [8] utilized bilateral filtering with grayscale probability modeling, and Rong et al. [9] introduced a conditional diffusion model based on adaptive image patches. Although these methods are computationally efficient, they are often sensitive to parameter selection and complex clutter.

Visual attention mechanism-based methods enhance salient target regions using local contrast or gradient information. Representative methods include DoG [10] and LCM [11]. To further improve robustness, Qiu et al. [12] proposed the Globally Sparse-Weighted LCM (GSWLCM) by integrating global and local features with adaptive thresholding. Kou et al. [13] combined density peak clustering with local contrast computation, while Xie et al. [14] introduced MDCM using second-order derivatives and intensity information. He et al. [15] proposed VDWTLCM with a three-layer sliding window structure for multi-scale detection, and Li et al. [16] developed CGDM for heterogeneous and cluttered backgrounds. These methods can effectively enhance small targets. However, these methods remain susceptible to interference from salient non-target structures such as bright edges and sea clutter.

Sparse and low-rank representation-based methods assume that the background exhibits low-rank structure, while targets correspond to sparse components. Representative approaches include the infrared patch-image (IPI) method [17]. Hu et al. [18] proposed DMSnet with a nested attention architecture and double-step pyramid fusion module. Lu et al. [19] developed a spatial–temporal feature fusion tensor (STFFT) model for low-rank and sparse decomposition, while Zhang et al. [20] introduced the Interpretation Weighted Sparse (IWS) method by combining structure tensor enhancement with clutter suppression. Although these methods provide strong target–background separation capability, they generally suffer from relatively high computational complexity.

Deep learning-based methods have achieved significant progress in infrared small-target detection [21] by learning discriminative feature representations from large-scale annotated datasets. Representative methods include RISTDNet [22], YOLO-Security [23], spatiotemporal feature fusion networks [24], improved U-Net frameworks [25,26], GEFPN [27], SLA-Net [28], and YOLO-ssboat [29]. These methods enhance multi-scale feature extraction, attention modeling, and feature fusion to improve small-target ship detection in complex maritime environments. However, their performance generally depends on large annotated datasets and high computational cost, which may limit generalization and real-time applicability under low-SNR maritime conditions.

Compared with single-frame approaches, multi-frame small-target detection exploits temporal continuity across consecutive image frames to improve robustness and suppress false alarms. Existing multi-frame methods are generally categorized into detect-before-track (DBT) and track-before-detect (TBD) frameworks.

DBT methods first perform candidate target extraction in individual frames and then establish target trajectories through inter-frame association. Representative approaches include the low-threshold multi-frame detection method proposed by Buzzi et al. [30], MJDTM [31], and appearance-stability-based maritime target detection [32]. These methods are computationally efficient and relatively easy to implement; however, their performance strongly depends on the reliability of the initial single-frame detection results.

In contrast, TBD methods prioritize temporal trajectory accumulation before final target detection. Representative approaches include wavelet-based TBD [33], SFA-PF-TBD [34], dense-optical-flow-based motion detection [35], Bayesian estimation frameworks [36], and candidate-graph-based trajectory extraction [37]. Although TBD methods can improve weak-target detection performance, they generally involve higher computational complexity and may become unstable under prolonged occlusion or target disappearance.

In summary, existing single-frame detection methods often suffer from sensitivity to complex maritime clutter, high computational complexity, or limited generalization under low-SNR conditions. Although multi-frame methods can exploit temporal information to improve robustness, DBT frameworks rely heavily on the accuracy of initial single-frame detection, while TBD frameworks may face challenges in computational efficiency and tracking stability. Therefore, achieving robust and accurate small-target ship detection in complex sea–sky environments remains a challenging problem.

**Figure 1:**
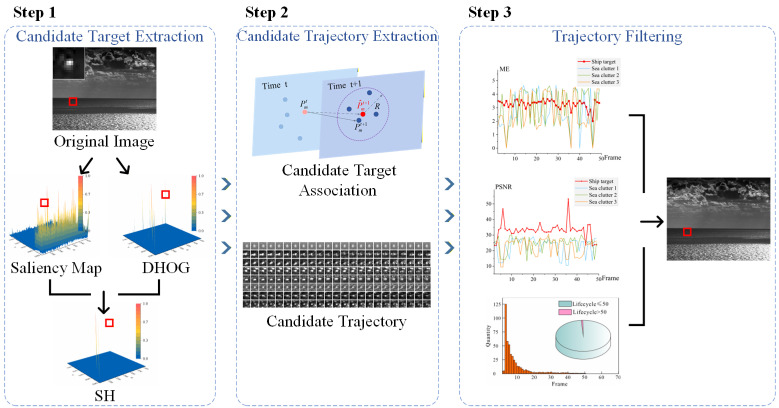
Schematic illustration of the proposed method pipeline.

Motivated by these limitations, this paper proposes a multi-frame spatiotemporal feature fusion framework for small-target ship detection. By jointly exploiting the spatial structural characteristics and temporal motion consistency of targets, the proposed method enhances detection robustness in complex sea–sky backgrounds. Specifically, the overall schematic pipeline of our proposed method is illustrated in Figure 1. As shown, our framework consists of three sequential core stages. First, in the “Candidate Target Extraction” stage, the original input image is processed by multi-scale HOG difference operation and saliency detection, to generate the fusion feature map and suppress complex sea–sky background clutter, and finally obtain reliable potential small-target candidates. Then, in the “Candidate Trajectory Extraction” stage, inverse optical flow matching and candidate target association are performed across consecutive multi frames, to link the detected candidates and construct complete candidate target trajectories over time. Finally, in the “Trajectory Filtering” stage, multi-feature joint filtering strategy is applied to evaluate and screen all candidate trajectories, eliminate false alarms, and output the final accurate small ship target detection result.

The remainder of this paper is organized as follows: Section 2 introduces the computational models of image gradients and Histograms of Oriented Gradients. Section 3 presents the proposed method, including the candidate target extraction based on DHOG-driven saliency detection, the trajectory extraction via inverse optical flow matching, and the multi-feature trajectory filtering strategy. Section 4 evaluates the effectiveness of the proposed approach through extensive experiments. Section 5 concludes the paper.

## 2. Theoretical Foundations

### 2.1. Calculation of Image Gradients

The image gradient characterizes the rate of change in pixel intensity within an image. The gradient magnitude reflects the degree of variation in pixel values, while the gradient direction indicates the orientation of this variation. At a given point ${\left(x,y\right)}^{T}$ in image $f\left(x,y\right)$, the gradient can be formally expressed as $\nabla f\left(x,y\right)$ in Equation (1):

(1) $$\nabla f\left(x,y\right)={\left[\begin{array}{cc} G_{x} & G_{y} \end{array}\right]}^{T}={\left[\begin{array}{cc} \frac{\partial f}{\partial x} & \frac{\partial f}{\partial y} \end{array}\right]}^{T},$$

where $G_{x}$ denotes the gradient component along the *x* direction, and $G_{y}$ denotes the gradient component along the *y* direction. Accordingly, the gradient vector can be constructed based on its magnitude $\left|\nabla f\left(x,y\right)\right|$ defined by Equation (2) and direction defined by Equation (3):

(2) $$\left|\nabla f\left(x,y\right)\right|=mag\left(\nabla f\left(x,y\right)\right)=\sqrt{{G_{x}}^{2}+{G_{y}}^{2}},$$

(3) ϕx,y=arctanGyGyGxGx

Image gradients play a fundamental role in describing structural features such as edges and textures. They are widely employed in numerous computer vision tasks, including corner detection, saliency estimation, image enhancement, object detection, and object tracking. By capturing local intensity variations, gradient-based representations provide essential information for subsequent image analysis and processing.

### 2.2. Calculation of Image Gradient Histogram

The Histogram of Oriented Gradients (HOG) [38], introduced by French researchers Navneet Dalal and Bill Triggs at the IEEE Computer Society Conference on Computer Vision and Pattern Recognition (CVPR 2005), is a widely used feature descriptor for representing local texture and shape information in images [39]. HOG has become a standard technique in computer vision and pattern recognition due to its robustness and effectiveness.

The core idea of HOG is to compute image gradients, group them within local regions, and represent each region by a histogram of gradient orientations. The computational procedure is described as follows.

An image can be regarded as a collection of discrete pixels. First, directional gradients are obtained by convolving the image along the horizontal and vertical directions using convolution kernels $w=\left[\begin{array}{ccc} -1 & 0 & 1 \end{array}\right]$ and $w={\left[\begin{array}{ccc} -1 & 0 & 1 \end{array}\right]}^{T}$, respectively. Subsequently, for each pixel, the gradient magnitude and orientation are computed using Equations (2) and (3). Directly extracting gradient features at the pixel level is sensitive to noise and computationally expensive. Therefore, the image is divided into smaller spatial regions, referred to as cells, to construct local feature descriptors. This strategy enhances robustness while reducing sensitivity to local noise. As an illustrative example, consider a cell of size 7×7.

**Figure 2:**
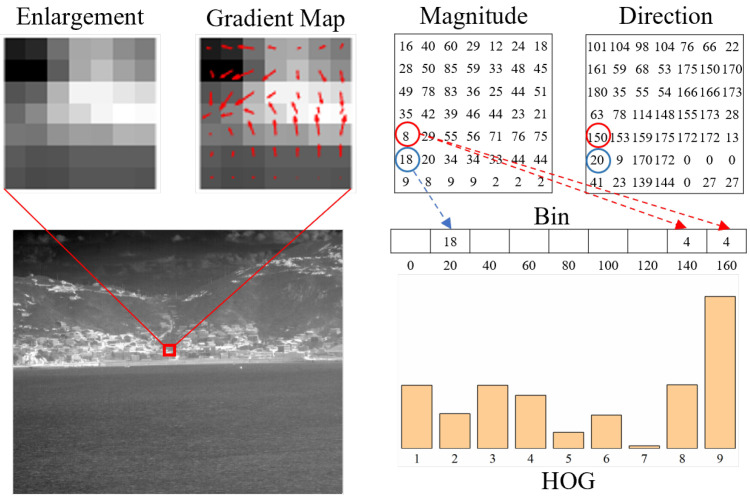
HOG calculation diagram. The Gradient Map, arrow direction denotes gradient orientation and arrow length represents gradient magnitude.

As shown in Figure 2, a 7×7 cell is selected from the image. The corresponding magnified region and its gradient maps are presented above the original image. For each pixel within the cell, the gradient magnitude and orientation are calculated, resulting in magnitude and orientation maps. The gradient orientations are then quantized into *N* equal bins within the range $\left[0,\pi \right]$, dividing the orientation space into several discrete intervals. The value of each bin is obtained by accumulating the gradient magnitudes of all pixels whose orientations fall within the corresponding interval. For example, when N=9, the orientation range is divided into 9 bins, each spanning 20°. Each pixel contributes to the histogram through a voting process. For instance, consider a pixel marked by a blue circle with a gradient magnitude of 18 and an orientation of 20°. Since 20° falls within the second bin, the value of that bin is incremented by 18. Similarly, for a pixel marked by a red circle with a gradient magnitude of 8 and an orientation of 150°, the magnitude is distributed proportionally between the two adjacent bins (e.g., 140–160°) according to a weighted voting scheme. In this case, each bin receives a value of 4. When the orientation lies near the boundary (e.g., between 160° and 180°), the magnitude is proportionally distributed between the 160° bin and the 0° bin to ensure continuity. After aggregating the contributions from all pixels within the cell, a histogram of oriented gradients is obtained. The resulting HOG descriptor for the cell is a feature vector of length *N*, representing the distribution of local gradient orientations.

## 3. Our Method

### 3.1. Saliency Map Computation Based on the Log Spectrum

Human visual saliency [40] is generally regarded as a pre-attentive process in which human observers naturally focus on visually distinctive regions within a scene. These salient regions often correspond to important objects or informative structures. Attention can be directed toward task-relevant targets by performing saliency analysis on images, thereby improving both the accuracy and efficiency of subsequent target detection. Consequently, saliency-based methods have been widely applied in target detection tasks [41]. Compared with conventional feature extraction techniques, saliency-based approaches are particularly effective in suppressing background interference. In complex environments-such as cluttered terrains or cluttered cloud background scenes-saliency estimation helps attenuate irrelevant background structures, thus enhancing detection robustness and reliability. This makes saliency analysis highly valuable in image processing applications [42].

In complex backgrounds, infrared small targets are frequently embedded in noise and clutter. However, their intensity typically differs from that of the surrounding regions, either being significantly brighter or darker. Such contrast characteristics make them more likely to attract human visual attention. Empirical studies have shown that when computing the logarithmic spectrum and corresponding frequency curves for a certain number of natural images, the Log–Log spectral distribution tends to exhibit an approximately linear trend [43]. By subtracting the log spectrum from the original log spectrum and subsequently performing an inverse Fourier transform, the residual spectrum can be obtained, which highlights salient regions and improves the signal-to-cultter ratio.

Assume that the input infrared image is denoted as $I\left(x,y\right)$. According to Equation (4), its Fourier spectrum can be obtained via the two-dimensional Discrete Fourier Transform (2D-DFT):

(4) $$f\left(x,y\right)=F\left[I\left(x,y\right)\right]=\sum_{n_{x}=0}^{N_{x}-1}\sum_{n_{y}=0}^{N_{y}-1}I\left(n_{x},n_{y}\right)e^{-j2\pi \left(\frac{x}{N_{x}}n_{x}+\frac{y}{N_{y}}n_{y}\right)},$$

where $F\left[\cdot \right]$ denotes the 2D-DFT operator, comprising the magnitude spectrum $A\left(x,y\right)$ and the phase spectrum $P\left(x,y\right)$:

(5) $$A\left(x,y\right)=Amplitude\left(F\left[I\left(x,y\right)\right]\right),$$

(6) $$P\left(x,y\right)=Phrase\left(F\left[I\left(x,y\right)\right]\right),$$

where $Amplitude\left(\cdot \right)$ represents the magnitude operator and $Phrase\left(\cdot \right)$ denotes the phase operator derived from Euler’s Equation.

The log spectrum is computed as Equation (7):

(7) $$L\left(x,y\right)=\log\left(A\left(x,y\right)\right).$$

A mean filter $H_{n}\left(x,y\right)$ is then applied to the log spectrum to obtain a smoothed representation. The residual spectrum *R* is calculated by subtracting the smoothed log spectrum from the original log spectrum, as shown in Equation (8):

(8) $$R\left(x,y\right)=L\left(x,y\right)-H_{n}\left(x,y\right)*L\left(x,y\right).$$

Finally, the saliency map $S\left(x,y\right)$ is obtained by performing the inverse Discrete Fourier Transform (IDFT) on the residual spectrum combined with the original phase information, followed by Gaussian smoothing, as shown in Equation (9):

(9) $$S\left(x,y\right)=Ga\left(x,y\right)*{\left|F^{-1}\left[\exp\left(R\left(x,y\right)+iP\left(x,y\right)\right)\right]\right|}^{2},$$

where $F^{-1}\left[\cdot \right]$ denotes the inverse 2D-DFT operator, $Ga\left(x,y\right)$ represents Gaussian filtering, and *i* is the imaginary unit.

Through the above procedure, the saliency map based on the log spectrum is obtained.

### 3.2. Candidate Object Extraction Based on HOG Differences

#### 3.2.1. Differential Histogram of Oriented Gradient Algorithm

After saliency detection, salient regions are enhanced while most redundant background information is suppressed. However, saliency maps may still contain both true targets and noise, particularly in complex backgrounds with strong edges or textured structures. Therefore, an effective discrimination mechanism is required to distinguish targets from background clutter.

To address this issue, we propose a Differential Histogram of Oriented Gradients (DHOG) algorithm, which differentiates targets from background regions by analyzing disparities in HOG features.

**Figure 3:**
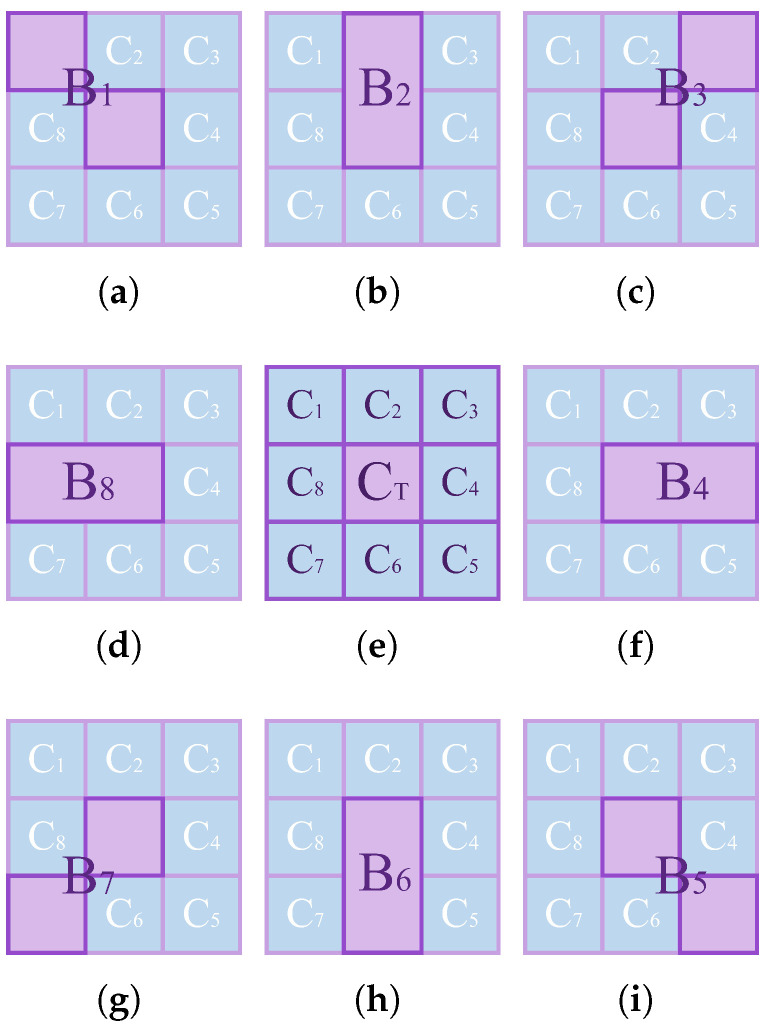
The structure of a sliding window. (**a**) Block $B_{1}$. (**b**) Block $B_{2}$. (**c**) Block $B_{3}$. (**d**) Block $B_{8}$. (**e**) Central cell $C_{T}$. (**f**) Block $B_{4}$. (**g**) Block $B_{7}$. (**h**) Block $B_{6}$. (**i**) Block $B_{5}$.

First, a sliding window is constructed to scan the entire image from left to right and from top to bottom, as illustrated in Figure 3e. The sliding window consists of 3×3 cells of equal size. The central cell represents the candidate target region, denoted as $C_{T}$, while the eight surrounding cells correspond to background regions, commencing sequentially from the top-left corner and labeled clockwise as $C_{1}$ to $C_{8}$. The size of each cell is selected to approximately match the expected target size (e.g., (7×7) pixels). To measure local contrast within the sliding window more precisely, the window is partitioned into block regions. Each block is formed by combining the central cell with one of the surrounding background cells, resulting in eight block regions in total. For example, the combination of the central cell and the top-left background cell forms the first block, denoted as $B_{1}$; the remaining blocks are defined analogously.

The sliding window is moved across the image with a predefined step size NTNT22 in both horizontal and vertical directions. For each cell within the sliding window—including the central cell and the eight surrounding cells—the HOG feature vector is computed. Let $H_{C_{T}}$ denote the HOG feature of the central cell and $H_{C_{1}}$ to $H_{C_{8}}$ denote the HOG feature of the eight background cells. The HOG difference for each block is defined as $d\left(B_{i}\right)$:

(10) $$\begin{array}{cc} d\left(B_{i}\right)={∥H_{C_{T}}-H_{C_{i}}∥}_{2} & \left(i=1\ldots 8\right) \end{array},$$

where ${∥\cdot ∥}_{2}$ represents the ($L_{2}$) norm.

To further enhance target responses while suppressing background clutter and noise, we introduce the Differential Histogram of Oriented Gradients (DHOG) operator. When the sliding window is positioned at a given location $\left(m,n\right)$, the DHOG response of the central cell is defined as Equation (11):

(11) $$DHOG_{\left(m,n\right)}=\bar{d}\left(B_{i}\right)-\mathrm{var}\left[d\left(B_{i}\right)\right],$$

where $\bar{d}\left(B_{i}\right)$ denotes the mean of the HOG difference values across the eight block regions:

(12) $$\bar{d}\left(B_{i}\right)=\frac{1}{8}\sum_{i=1}^{8}d\left(B_{i}\right),$$

and $\mathrm{var}\left[d\left(B_{i}\right)\right]$ denotes their variance, as shown in Equation (13):

(13) $$\mathrm{var}\left[d\left(B_{i}\right)\right]=\frac{\sum_{i=1}^{8}{\left[d\left(B_{i}\right)-\bar{d}\left(B_{i}\right)\right]}^{2}}{8}.$$

By jointly considering the mean and variance of local HOG differences, the DHOG operator effectively emphasizes structural discrepancies between targets and their surrounding background, thereby improving candidate object extraction performance.

The idea of using traditional algorithms to extract features for detection assistance has been studied in ship detection. Ke et al. [44] proposed a Laplace and LBP feature guided SAR ship detection method with an adaptive feature enhancement block. Compared with this method, a brief discussion of how the proposed DHOG approach differs. Key distinctions are as follows: (1) our method operates on infrared (rather than SAR or optical) imagery; (2) we exploit HOG orientation histograms rather than LBP or Laplacian responses, making the method insensitive to absolute intensity but sensitive to local gradient structure; and (3) DHOG is applied at the candidate extraction stage as a lightweight filter, whereas the cited methods use similar features as auxiliary inputs to deep networks. Our paper further introduces spatiotemporal features to better exploit the information from sequential images for extracting moving ship targets.

#### 3.2.2. Analysis of DHOG

(1) Analysis of Target’s DHOG Characteristics

The experimental dataset used in this study consists exclusively of infrared (IR) images; therefore, the following analysis focuses on infrared imaging mechanisms and target characteristics. All objects with temperatures above absolute zero (−273.15 °C) emit infrared radiation, and the emitted energy is positively correlated with temperature. Infrared radiation is a form of electromagnetic wave, typically with wavelengths ranging from 0.78 μm to 1000 μm, corresponding to a frequency range of approximately 300 THz to 400 THz. In infrared imaging systems, temperature differences between objects and their surroundings are converted into grayscale variations in the captured image.

As man-made objects, ship targets generally exhibit higher temperatures than their natural surroundings. This observation also applies to other artificial targets, such as vehicles, aircraft, and missiles, which often contain high-temperature components (e.g., engines or heat sources). These components produce stronger infrared radiation, resulting in higher grayscale values compared with the background. However, under certain conditions—such as the presence of cooling systems, long imaging distances, or low ambient temperatures—the target may appear darker than the surrounding background. Such targets are referred to as dark targets. The thermal contrast between target and background leads to distinctive grayscale distributions in infrared images. Target grayscale values tend to concentrate within a relatively narrow range, whereas background grayscale values are typically more dispersed. Furthermore, atmospheric refraction, optical defocusing, and lens aberrations often cause small infrared targets to appear as approximately circular Gaussian-like point sources. Numerous studies have shown that small targets can be effectively modeled using a two-dimensional Gaussian point spread function (PSF), expressed as Equation (14):

(14) $$f\left(x,y\right)=\gamma \cdot \exp\left\{-\frac{1}{2}\left[{\left(\frac{x-x_{0}}{\sigma_{x}}\right)}^{2}+{\left(\frac{y-y_{0}}{\sigma_{y}}\right)}^{2}\right]\right\},$$

where γ denotes the peak amplitude of the target; $\sigma_{x}$ and $\sigma_{y}$ represent the dispersion in the horizontal *x* and vertical *y* directions, respectively; and $\left(x_{0},y_{0}\right)$ denotes the target center. Smaller values of $\sigma_{x}$ and $\sigma_{y}$ indicate a more compact energy distribution.

**Figure 4:**
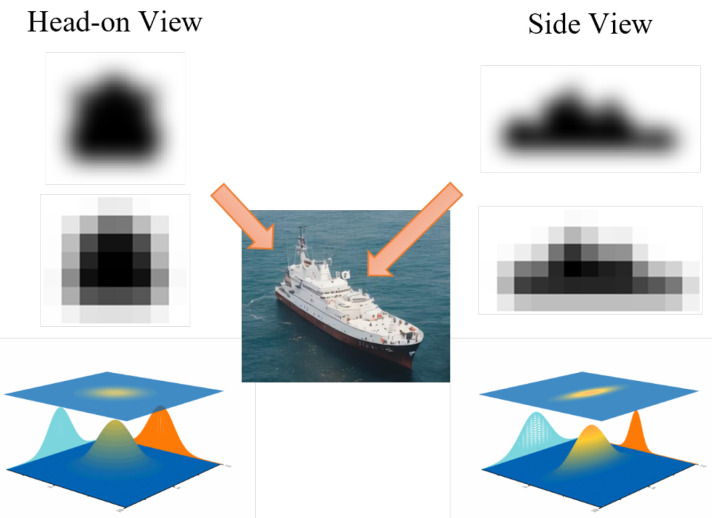
Analysis of small ship targets from different perspectives. It displays point spread effect, discrete digital features and approximated 3D energy distributions of ships under head-on and side views. In the approximated 3D energy distributions, warmer red color corresponds to higher energy near peak values.

For small ship targets, geometric and observational factors introduce anisotropy in the energy distribution. In particular, variations in viewing angle lead to differences in dispersion in the horizontal and vertical directions, which constitute a distinctive characteristic of ship targets. As illustrated in Figure 4, the point spread effect, digital discretization, and approximate three-dimensional energy distributions differ depending on whether the ship is observed from a head-on or side-view perspective.

After digital discretization, a ship observed from a head-on perspective typically appears as an approximately isotropic point target. The Gaussian profiles projected along the *x*- and *y*-axes exhibit similar amplitudes and dispersions, which is consistent with the commonly adopted isotropic Gaussian model in many existing algorithms. In contrast, when the ship is observed from a side view, the target appears elongated and approximately elliptical. Although the peak amplitude remains similar in both directions, the dispersions $\sigma_{x}$ and $\sigma_{y}$ differ, reflecting the anisotropic structure of the ship.

**Figure 5:**
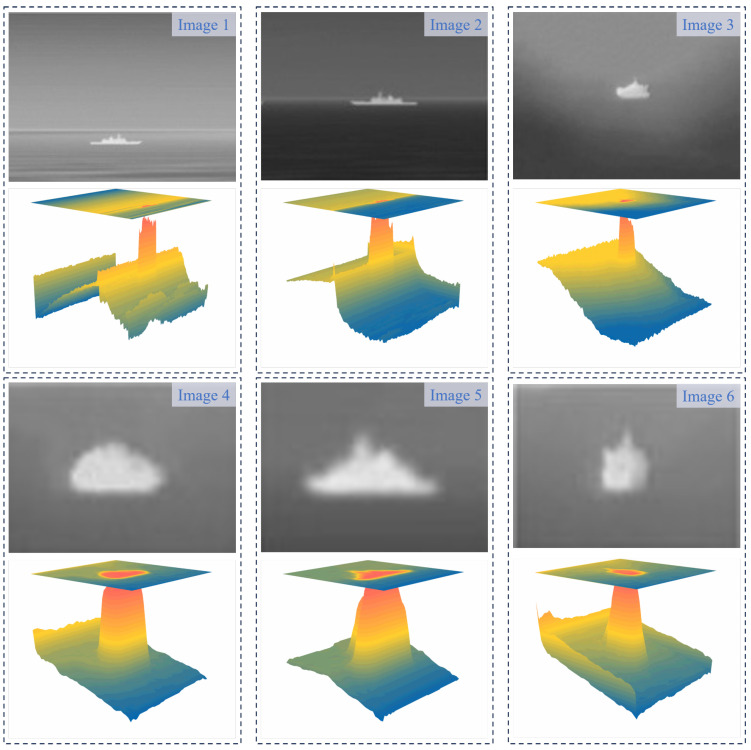
Several ship targets and their corresponding three-dimensional energy distributions. Redder regions stand for higher energy close to the peak.

Figure 5 presents several ship targets and their corresponding three-dimensional energy distributions, redder regions stand for higher energy close to the peak. The target energy profiles are fitted using the Gaussian model described above, and the estimated parameters γ, $\sigma_{x}$ and $\sigma_{y}$ are summarized in Table 1. For example, the parameters in the first row of Table 1 are the Gaussian model fitting results for the target shown in Image 1 of Figure 5.

Based on the fitting results and the measured target sizes in the images, parameter γ is primarily associated with the grayscale contrast between the ship target and the background. Parameters $\sigma_{x}$ and $\sigma_{y}$ characterize the spatial energy distribution along the horizontal and vertical directions, respectively, and are influenced by the target orientation and the angle between the ship and the camera optical axis. Specifically, under side-view observation, $\sigma_{x}$ is approximately one-sixth of the ship length, while $\sigma_{y}$ is approximately one-sixth of the ship height. Their ratio reflects the length-to-height ratio of the ship. Under head-on observation, $\sigma_{x}$ is approximately one-sixth of the ship width and $\sigma_{y}$ is approximately one-sixth of the ship height; their ratio corresponds to the width-to-height ratio. For intermediate viewing angles, $\sigma_{x}$ typically lies between one-sixth of the ship width and one-sixth of the ship length, whereas $\sigma_{y}$ remains close to one-sixth of the ship height.

These observations are consistent with the energy distribution patterns shown in Figure 4. Therefore, for head-on views, a circular point template is appropriate for target modeling, whereas for side-view observations, an elliptical template provides a more accurate representation. In both cases, the DHOG features extracted from these target templates differ significantly from those of the surrounding background.

Based on this analysis, the following section examines the DHOG characteristics of circular bright and dark targets under front-view conditions, as well as elliptical targets observed from side-view perspectives.

(a) Circular Bright Target

**Figure 6:**
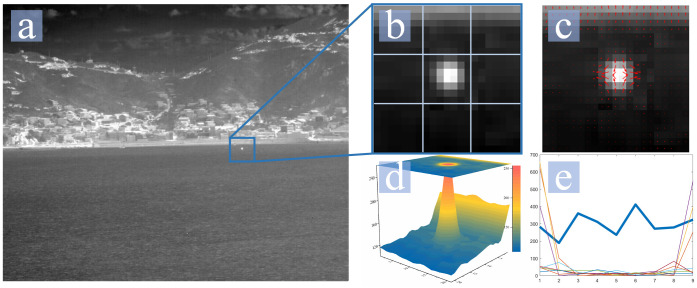
HOG difference analysis map of the bright target. (**a**) Original image. (**b**) Grayscale distribution of cropped regions divided into nine cells. (**c**) Gradient vector field where arrow direction and length indicate gradient orientation and magnitude respectively. (**d**) 3D visualization of cropped areas. (**e**) HOG curves of nine cells.

Figure 6a presents an infrared image with sea and sky as the background, in which small moving ship targets appear as bright point-like objects. Owing to their relatively strong thermal radiation, ship targets typically manifest as bright regions in infrared imagery. At long imaging distances or under head-on viewing conditions, a ship target may appear approximately circular and point-like. A 21×21 pixel region centered at the target centroid is magnified in Figure 6b, and its three-dimensional intensity distribution is shown in Figure 6d. The target can be well approximated by a two-dimensional Gaussian surface, where the maximum grayscale value occurs at the center and gradually attenuates toward the periphery with approximately isotropic decay rates.

Figure 6c illustrates the gradient vector field within this local region. The arrow length represents the gradient magnitude at the corresponding pixel (located at the arrow tail), while the arrow direction indicates the orientation of the image gradient. As shown in Figure 3, the region is divided into 3×3 cells of equal size, and the HOG descriptor is computed for each cell. With an orientation interval of 20°, each cell is represented by a 9-dimensional HOG vector. The corresponding HOG distributions are plotted in Figure 6e, where the thick blue solid line denotes the HOG of the central cell and the thinner colored lines represent the HOG curves of the surrounding background cells.

It can be observed that the HOG response of the central cell is significantly higher than that of the background cells. This is because the target region exhibits a pronounced grayscale gradient from the center toward the surrounding area, as described by the quasi-two-dimensional Gaussian model. The resulting gradient magnitudes are relatively large and distributed in a nearly uniform manner across all directions, leading to a strong and evenly distributed HOG response in the central cell.

(b) Circular Dark Target

Although ship targets are generally bright in infrared images, certain scenarios may lead to lower grayscale values relative to the background. This situation may arise when the background is comparatively brighter (e.g., due to high-altitude clouds) or when the target includes cooling mechanisms. In such cases, the target appears as a dark circular point and is referred to as a dark target. The proposed algorithm is equally applicable to dark targets; therefore, their characteristics are also analyzed.

**Figure 7:**
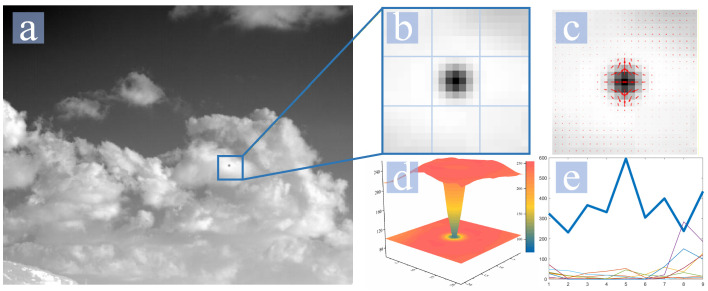
HOG difference analysis map of the dark target. (**a**) Original image. (**b**) Grayscale distribution of cropped regions divided into nine cells. (**c**) Gradient vector field where arrow direction and length indicate gradient orientation and magnitude respectively. (**d**) 3D visualization of cropped areas. (**e**) HOG curves of nine cells.

Figure 7a shows an infrared image with the sky as the background, containing altocumulus clouds with relatively uniform yet bright clustered textures. When a ship target enters such a region, its relative grayscale becomes lower than that of the background, resulting in a dark target appearance. A 21×21 pixel region centered at the target centroid is extracted for analysis. Its grayscale distribution and three-dimensional representation are shown in Figure 7b and Figure 7d, respectively. The target still conforms approximately to a two-dimensional Gaussian model; however, in this case, the grayscale value at the center is lower than that of the surrounding area. The intensity gradually increases from the center toward the periphery with approximately isotropic variation. The gradient vector field is shown in Figure 7c. It can be observed that gradient vectors in the outer background region are shorter and exhibit diverse orientations, whereas vectors within the central target cell are longer and predominantly point toward the center, reflecting stronger and more coherent gradient structures.

After computing the HOG descriptors for the nine cells in Figure 7b, the resulting HOG curves are shown in Figure 7e. Similar to the bright target case, the HOG of the central cell indicated by the thick blue line is significantly higher than that of the surrounding cells. Moreover, since the dark target closely approximates an ideal two-dimensional Gaussian distribution, the HOG response in the central cell is relatively uniform across orientation bins.

(c) Elliptical Bright Target

**Figure 8:**
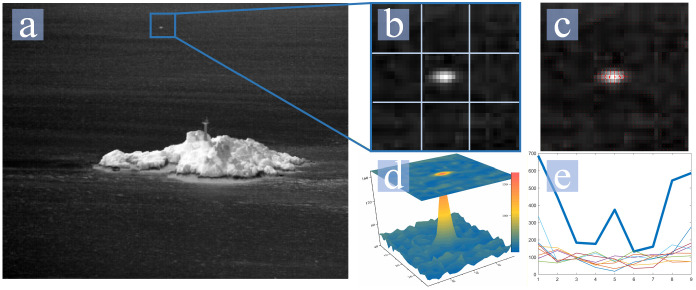
HOG difference analysis map of an elliptical target. (**a**) Original image. (**b**) Grayscale distribution of cropped regions divided into nine cells. (**c**) Gradient vector field where arrow direction and length indicate gradient orientation and magnitude respectively. (**d**) 3D visualization of cropped areas. (**e**) HOG curves of nine cells.

Figure 8a presents an infrared image of a ship target observed from a side-view perspective. As shown in the magnified local region in Figure 8b, the target exhibits an elliptical shape with high grayscale values. Its three-dimensional intensity distribution is shown in Figure 8d. The centroid corresponds to the maximum grayscale value, which gradually attenuates toward the boundary. Unlike the circular case, the attenuation rates differ along different directions. Specifically, the decay is slower along the longitudinal direction of the ship, that is along the direction of motion, and faster along the transverse direction, resulting in an anisotropic energy distribution. Figure 8c illustrates the gradient vector field in the target region, and Figure 8e shows the HOG curves for the nine cells. It can be observed that orientation bins corresponding to the longitudinal direction of the ship (approximately 0–20° and 160–180°) exhibit relatively high responses, with the maximum typically occurring in bin 1 or bin 9. This phenomenon is consistent with the anisotropic structure of the elliptical target, where dominant gradients align with the principal axes of the ship. It can be observed that orientation bins near and exhibit relatively high responses, with the maximum typically occurring near. This phenomenon is consistent with the anisotropic structure of the elliptical target, where dominant gradients align with the principal axes of the ship.

In contrast, the HOG responses in the surrounding background cells are generally weaker and irregularly distributed, making it difficult to characterize them with a consistent structural pattern. A more detailed analysis of background behavior will be provided in the Section 3.2.2 part (2).

(2) Analysis of Target’s DHOG Characteristics

Ship target detection in sea–sky scenes is often challenged by various forms of background clutter. When a target appears against a relatively homogeneous background, the grayscale contrast between the target and its surroundings is pronounced, resulting in minimal interference during detection. However, in practical scenarios, infrared images frequently contain complex and heterogeneous backgrounds, which can generate false alarms and significantly reduce the signal-to-clutter ratio (SCR) [45], thereby increasing detection difficulty. Typical background types include flat sea surfaces, tree-lined or mountainous coastal regions, buildings, clouds, and turbulent sea waves. The DHOG characteristics of each background type are analyzed below.

(a) Flat Backgrounds

**Figure 9:**
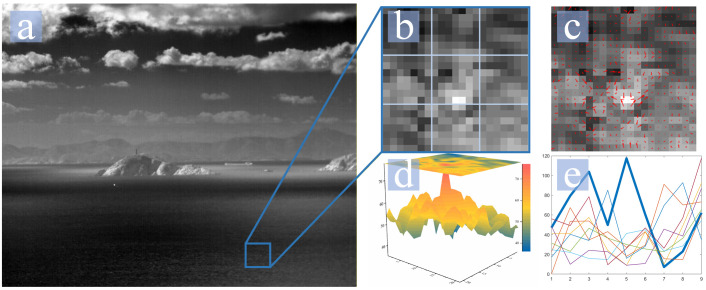
HOG difference analysis map with the flat background. (**a**) Original image. (**b**) Grayscale distribution of cropped regions divided into nine cells. (**c**) Gradient vector field where arrow direction and length indicate gradient orientation and magnitude respectively. (**d**) 3D visualization of cropped areas. (**e**) HOG curves of nine cells.

Flat background regions exhibit minimal grayscale variation, as illustrated by the calm sea surface in Figure 9a. Under such conditions, the sea remains relatively smooth with limited wave activity. Due to the absorption and scattering properties of seawater, the emitted infrared radiation is relatively weak. Moreover, the temperature distribution across the sea surface is generally uniform, resulting in the absence of significant thermal gradients and an overall dark appearance in the infrared image. The grayscale distribution is shown in Figure 9b, and the corresponding three-dimensional representation is provided in Figure 9d. Pixel intensities in this region are consistently low, with only slight differences between adjacent pixels. Consequently, the grayscale difference between the central cell and its neighboring cells is negligible.

The gradient vector field in Figure 9c shows short arrows with irregular orientations, indicating weak and incoherent gradient structures. The HOG curves for the nine cells are presented in Figure 9e. The HOG curve of the central cell marked with bold blue line is embedded among the curves of the surrounding cells, and all curves exhibit similar trends. This indicates that the DHOG response in flat background regions is weak and lacks distinctive structural characteristics.

(b) Tree-Lined Backgrounds

**Figure 10:**
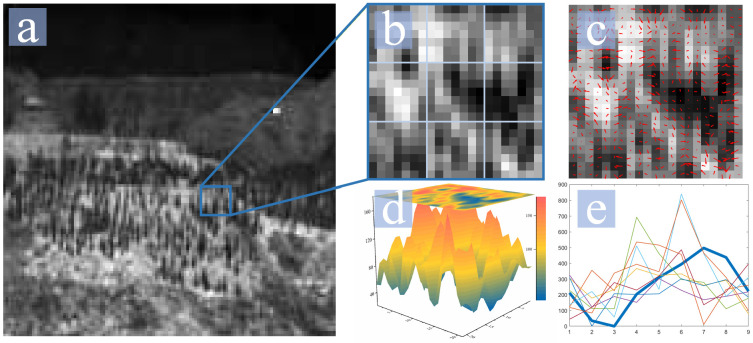
HOG difference analysis map with the tree-lined background. (**a**) Original image. (**b**) Grayscale distribution of cropped regions divided into nine cells. (**c**) Gradient vector field where arrow direction and length indicate gradient orientation and magnitude respectively. (**d**) 3D visualization of cropped areas. (**e**) HOG curves of nine cells.

In nearshore or island environments, backgrounds often include mountains, trees, and vegetation, which can produce strong interference in ship detection tasks. Vegetation typically emits relatively high levels of infrared radiation and may appear bright in the image, whereas mountainous terrain often exhibits lower surface temperatures and appears darker. In addition, trees and vegetation possess complex textures and structural patterns. Combined with shadowing, reflection, and scattering effects, these characteristics produce alternating bright and dark regions in the image. As shown in Figure 10a, mountainous areas may exhibit relatively uniform grayscale distributions, whereas vegetation regions display irregular and chaotic intensity variations, often generating localized bright spots that resemble small targets.

Due to variations in tree size and density, these bright spots may appear elongated or irregularly shaped. The gradient magnitude and orientation vary randomly from the center of such bright regions outward. This randomness leads to similar HOG distributions for both the central and surrounding cells, as shown in Figure 10e. Consequently, tree-lined backgrounds do not produce the structured and concentrated DHOG responses typically associated with true ship targets.

(c) Building Backgrounds

**Figure 11:**
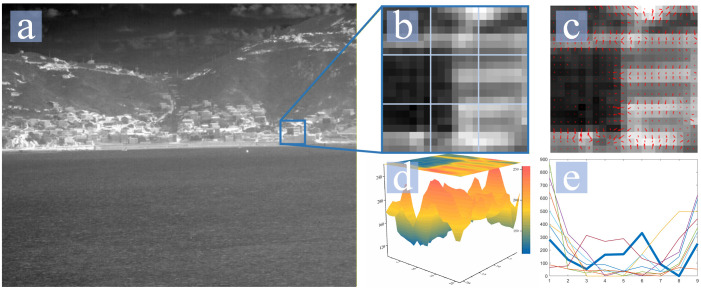
HOG difference analysis map with the building background. (**a**) Original image. (**b**) Grayscale distribution of cropped regions divided into nine cells. (**c**) Gradient vector field where arrow direction and length indicate gradient orientation and magnitude respectively. (**d**) 3D visualization of cropped areas. (**e**) HOG curves of nine cells.

In coastal scenes, building structures frequently appear in the background, as illustrated by the region marked in Figure 11a. Building surfaces partially reflect solar radiation, resulting in specific brightness patterns. However, differences in construction materials across building components lead to variations in thermal capacity and conductivity, causing spatially heterogeneous temperature distributions in infrared imagery. Furthermore, due to their structural thickness, buildings exhibit significant thermal inertia, meaning that temperature changes occur more gradually compared with vegetation. Nevertheless, sharp temperature gradients may appear along structural edges. As shown in Figure 11b, the edge region of a building displays a step-like grayscale distribution, with lower intensities on one side and higher intensities on the other. The gradient vector field in Figure 11c shows relatively long arrows near the edge, oriented toward regions of lower grayscale values. Cells located along the edge as cells $C_{T}$, $C_{2}$, and $C_{6}$, exhibit similar HOG distributions, with gradients concentrated around bin 5 corresponding to the edge normal direction. In contrast, HOG distributions in non-edge cells are relatively flat.

This directional concentration differs from the isotropic distribution observed in circular ship targets and the anisotropic yet coherent distribution of elliptical targets.

(d) Cloud Backgrounds

Clouds and fog exhibit diverse shapes and structural characteristics, resulting in significant variations in morphology, brightness, and texture. Thick and dense cumulus or stratus clouds often appear brighter, whereas thinner cirrus clouds tend to appear darker. In general, cloud tops are warmer and brighter, while cloud bases are cooler and darker. Cirrus clouds frequently exhibit filamentous or banded structures with fine textures.

**Figure 12:**
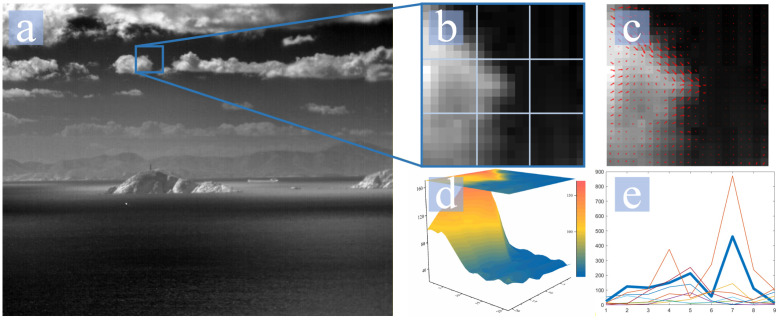
HOG difference analysis map with the cloud background. (**a**) Original image. (**b**) Grayscale distribution of cropped regions divided into nine cells. (**c**) Gradient vector field where arrow direction and length indicate gradient orientation and magnitude respectively. (**d**) 3D visualization of cropped areas. (**e**) HOG curves of nine cells.

Figure 12a shows an infrared image with clouds as the dominant background. The central regions of large cloud clusters may exhibit relatively smooth textures and can sometimes be approximated as flat backgrounds. However, significant intensity fluctuations occur near cloud edges or within smaller cloud clusters. As shown in Figure 12b, grayscale values change abruptly in the direction perpendicular to the cloud boundary. The maximum image gradients are located along these edges. Consequently, the gradient orientations of cells $C_{T}$, $C_{2}$, and $C_{6}$, positioned near the edge are strongly aligned with the normal direction of the cloud boundary, resulting in pronounced peaks in the corresponding HOG bins.

This highly directional gradient distribution differs from the more uniformly distributed gradients of Gaussian-like ship targets.

(e) Turbulent Sea Wave Backgrounds

**Figure 13:**
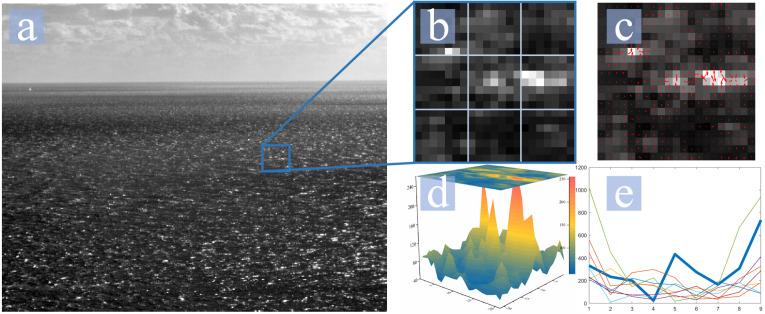
HOG difference analysis map with the turbulent sea wave background. (**a**) Original image. (**b**) Grayscale distribution of cropped regions divided into nine cells. (**c**) Gradient vector field where arrow direction and length indicate gradient orientation and magnitude respectively. (**d**) 3D visualization of cropped areas. (**e**) HOG curves of nine cells.

In contrast to the calm sea surface described earlier, variations in wind speed and sea temperature can produce turbulent sea conditions characterized by pronounced fluctuations and complex textures, as shown in Figure 13a. Solar radiation interacting with wave crests generates specular and diffuse reflections, producing alternating bright and dark streaks or spots in the infrared image. Turbulent mixing of seawater also induces localized temperature variations, leading to irregular brightness distributions. In the 21×21 region shown in Figure 13b, several bright point-like structures are visible. These structures can easily trigger false alarms, particularly when one appears within the central cell, producing a spike-like response in the HOG curve as shown in Figure 13e. However, these interfering bright spots vary in size and often deviate from an ideal isotropic two-dimensional Gaussian model. Their gradient structures lack the coherent and symmetric properties of true ship targets. As a result, although localized HOG responses may be elevated, the overall HOG patterns are not significantly distinguishable from those of neighboring background cells.

Overall, the above analysis demonstrates that background regions typically exhibit either weak, irregular, or highly directional gradient structures. In contrast, true ship targets produce coherent and structurally consistent HOG patterns. This distinction provides the theoretical foundation for the effectiveness of the proposed DHOG-based discrimination method.

#### 3.2.3. Multi-Scale Differential Histogram of Oriented Gradient Algorithm

In summary, the characteristics of small maritime targets can be described as follows: the target information is concentrated within a very limited number of pixels, and the targets typically lack clear contours and distinct shape features. The grayscale values within the target region are relatively uniform, and the contrast between the target and the surrounding background is generally low. In infrared imagery, such targets usually appear as bright or dark point-like structures or small elliptical regions. Therefore, the differences in the Histogram of Oriented Gradients (HOG) between the target region and the surrounding background can effectively distinguish the target from most background clutter. The candidate target extraction algorithm based on the Differential Histogram of Oriented Gradients (DHOG) can generate single-scale HOG maps. The detailed procedure is described in Algorithm A1.

Ideally, the cells illustrated in Figure 3 should have a size comparable to that of the target in order to achieve optimal detection performance when the central cell $C_{T}$ contains the target. However, in practical scenarios, the size of the target may vary due to differences in target distance, motion, and imaging conditions. A multi-scale design is introduced to address this issue. The proposed method utilizes gradient features derived from multi-scale HOG differences to perform saliency detection while effectively suppressing background clutter.

Specifically, $l_{\max}$ different scales are considered. Cells with different sizes form sliding windows of corresponding dimensions and scan the entire image independently. For each pixel location, the maximum response among the different scales is selected as the final value of the multi-scale DHOG response map. The detailed procedure is described in Algorithm A2, where the input image has a size of m×n.

Subsequently, a fusion feature map SH is generated by multiplying the saliency map *S* with the multi-scale DHOG response map $\hat{H}$, which further enhances the target response:

(15) $$SH=S\times \hat{H}.$$

Finally, the target location is determined using an adaptive threshold, whose calculation is given in Equation (16):

(16) Th=μ+kσ,

where μ denotes the mean intensity of the image, σ represents the standard deviation of the image, and *k* is a scaling factor. Based on empirical engineering experience, *k* is typically set to 3.

Since the candidate targets obtained after binarization may vary in size and shape, the target region is further analyzed using a fixed-size neighborhood centered at the centroid of each candidate region. The centroid $\left[x_{c},y_{c}\right]$ is computed as Equation (17):

(17) $$\left\{\begin{array}{c} x_{c}=\sum_{i=1}^{M}l_{i}\cdot x_{i}/\sum_{i=1}^{M}l_{i}\\ y_{c}=\sum_{i=1}^{M}l_{i}\cdot y_{i}/\sum_{i=1}^{M}l_{i} \end{array}\right.,$$

where $\left[x_{i},y_{i}\right]$ denotes the coordinates of the i-th pixel in the region, $l_{i}$ represents the grayscale intensity of that pixel, and *M* is the total number of pixels within the region.

Based on the above procedure, the complete candidate target extraction process can be implemented as described in Algorithm A3.

The multi-scale Differential Histogram of Oriented Gradients (DHOG) algorithm is designed to adapt to multi-scale small ship targets in sea–sky backgrounds, and the selection of its key parameters is based on the statistical characteristics of the target size in the experimental dataset and the balance between detection performance and computational complexity. Specifically, the cell size is set to be similar to the minimum target size (3 × 3 pixels) in the dataset to ensure effective capture of target gradient features; the maximum number of scales (lmax) is set to 5, covering the target size range (3 × 3 to 15 × 15 pixels) in the experimental sequences. The candidate cell sizes are 3 × 3, 5 × 5, 7 × 7, 9 × 9, and 11 × 11 pixels, corresponding to the 5 scales respectively. The sliding step is fixed at NT/2 (half the cell size per scale) pixels to balance detection accuracy and computational efficiency, while the number of HOG bins is set to 9 (0–180°, interval of 20°), which is optimal for capturing the gradient orientation distribution of small ship targets. The sliding window size for candidate extraction is set to 3 × 3 cell window, covering the maximum target size in the dataset to avoid missing large-scale targets. The scale-wise performance differences are mainly reflected in the fact that small cell sizes (3 × 3, 5 × 5) are more sensitive to small targets (3 × 3–7 × 7 pixels), while large cell sizes (9 × 9, 11 × 11) perform better for relatively large targets (9 × 9–15 × 15 pixels); the 7 × 7 cell size achieves the best comprehensive performance for multi-scale targets.

To verify the impact of scale settings (i.e., $l_{max}$ and candidate cell sizes) on the detection performance of the DHOG algorithm, a sensitivity analysis is conducted by adjusting the number of scales and cell sizes while keeping other parameters unchanged. For the example experiments, the performance indicators (BSF, SCRG, and AUC) were recorded separately when $l_{max}$ was set to 3 (cell sizes: 3×3, 5×5, 7×7), 5 (cell sizes: 3×3, 5×5, 7×7, 9×9, 11×11), and 7 (cell sizes: 3×3 to 15×15 pixels). It was found that the computational complexity was the highest when $l_{max}$ was set to 7. When the cell size was too small, the gradient feature extraction became unstable, resulting in relatively low BSF and AUC. When the cell size was too large, it was difficult to detect small targets, leading to a decrease in SCRG and AUC. Therefore, $l_{max}=5$ and its corresponding candidate cell sizes (3×3 to 11×11 pixels) were ultimately selected as the optimal scale settings, which achieved a good balance between detection performance and computational complexity.

### 3.3. Candidate Trajectory Extraction Based on Inverse Optical Flow Matching

Background clutter that exhibits characteristics similar to those of the target may introduce interference during candidate point extraction from single-frame images. However, the grayscale distribution of a true target typically varies only slightly over time, and its spatial position demonstrates temporal continuity, without abrupt displacement between adjacent frames. Therefore, it is necessary to exploit multi-frame temporal correlation. By utilizing the temporal consistency of target motion, the true target location can be determined more reliably, thereby reducing the false alarm rate and improving detection accuracy. After suppressing background noise, candidate targets can be extracted through threshold-based segmentation. To avoid missing potential targets, a relatively low threshold is employed during the extraction stage, which may inevitably introduce additional false positives. Consequently, inter-frame information must be utilized to extract candidate motion trajectories, enabling the subsequent identification and filtering of true targets.

Matching local image regions between adjacent frames provides an effective way to establish correspondences between candidate targets over time. However, variations in illumination and imaging conditions may alter the appearance of the target, which complicates accurate inter-frame matching. To address this issue, this paper proposes a trajectory correlation algorithm based on inverse optical flow matching.

The concept of optical flow, first introduced by Gibson in 1950 [46], describes the apparent motion of image brightness patterns between consecutive frames. Optical flow methods estimate the motion velocity vector of each pixel by analyzing changes in grayscale intensity within the image sequence. Traditional optical flow approaches rely on the brightness constancy assumption [47] and approximate the motion field using directional derivatives of the image sequence. Based on the estimated changes in the motion field, moving objects and scene can subsequently be segmented.

Assume that the grayscale intensity of the image at time *k* is denoted by $I\left(x,y,t\right)$. Under the brightness constancy assumption of the target, the relationship holds as Equation (18):

(18) $$I\left(x,y,t\right)=I\left(x+dx,y+dy,t+dt\right).$$

Applying a first-order Taylor expansion yields Equation (19):

(19) $$I\left(x+dx,y+dy,t+dt\right)=I\left(x,y,t\right)+\frac{\partial I}{\partial x}dx+\frac{\partial I}{\partial y}dy+\frac{\partial I}{\partial t}dt.$$

Substituting Equation (19) into Equation (18) and simplifying leads to Equation (20):

(20) $$\frac{\partial I}{\partial x}dx+\frac{\partial I}{\partial y}dy+\frac{\partial I}{\partial t}dt=0.$$

Dividing both sides of the equation by dt gives Equation (21):

(21) $$\frac{\partial I}{\partial x}\frac{dx}{dt}+\frac{\partial I}{\partial y}\frac{dy}{dt}+\frac{\partial I}{\partial t}=0.$$

Rearranging the terms results in the optical flow constraint equation (22).

(22) $$\frac{\partial I}{\partial x}\vec{u}+\frac{\partial I}{\partial y}\vec{v}+\frac{\partial I}{\partial t}=0,$$

where $\vec{u}$ and $\vec{v}$ denote the motion velocities of the pixel along the *x*-axis and *y*-axis, respectively, which together constitute the optical flow vector of the pixel. ∂I∂I∂x∂x and ∂I∂I∂y∂y represent the directional derivatives of the image intensity along the *x*-axis and *y*-axis.

The above derivation shows that conventional optical flow methods estimate the motion field of pixels using known directional derivatives under the brightness constancy assumption. In contrast, the inverse optical flow approach adopted in this work performs the reverse operation; that is, it estimates the grayscale compensation term of pixels when the motion displacement is known.

Accordingly, Equation (19) can be rewritten as Equation (23):

(23) $$I\left(x+dx,y+dy,t+dt\right)=I\left(x,y,t\right)+\Delta I,$$

where ΔI represents the grayscale compensation term, calculated as Equation (24):

(24) $$\Delta I=\frac{\partial I}{\partial x}\vec{u}+\frac{\partial I}{\partial y}\vec{v}+\frac{\partial I}{\partial t}.$$

These equations indicate that when the directional derivatives and pixel displacement are known, the grayscale variation in a pixel can be estimated. By using this variation as a compensation term and adding it to the original grayscale value, the predicted grayscale intensity of the pixel in the next frame can be obtained.

If grayscale prediction is performed for all pixels within a candidate target region, a local predicted image patch of the candidate target can be generated. This predicted patch can then be used to perform matching with candidate targets in the subsequent frame, thereby establishing temporal correspondence and extracting candidate target trajectories.

**Figure 14:**
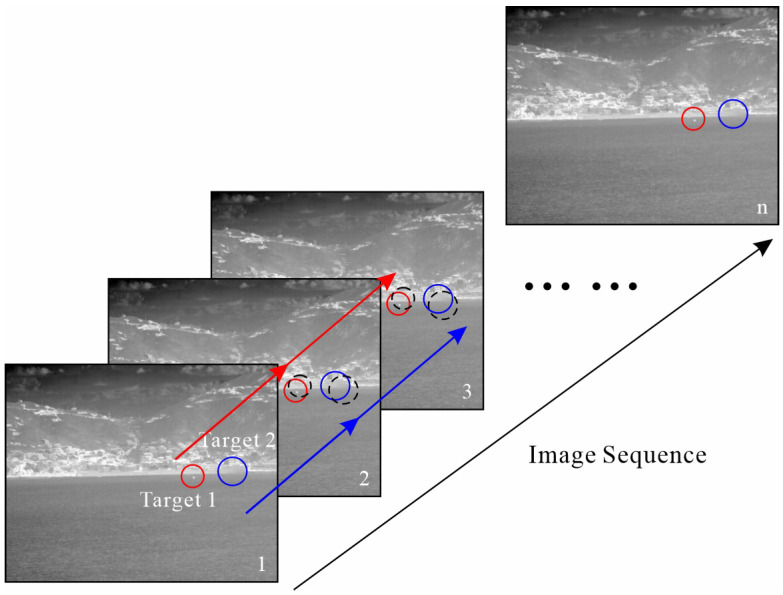
Schematic diagram of the principle of candidate trajectory extraction. Arrows denote the sequential order of image frames, dashed circles mark predicted candidate positions, and solid circles represent corrected positions, with red and blue highlighting two candidate target instances.

The overall principle of candidate trajectory extraction based on inverse optical flow matching is illustrated in Figure 14.

In the proposed method, Kalman filtering [48] is employed to estimate the motion pattern of the target and to update the motion state through grayscale matching between the predicted target intensity and the observed target intensity. The following section provides a detailed description of this procedure. When estimating the motion pattern of a target using Kalman filtering, the process begins with trajectory initialization. An initial trajectory is established for each candidate target detected in the first frame. Subsequently, the Kalman filtering algorithm is used to predict and estimate the states of candidate targets in subsequent frames. The predicted targets in the new frame are then matched with the existing motion trajectories using the proposed inverse optical flow matching algorithm, thereby updating the motion tracks. In the proposed method, Kalman filtering is employed to estimate the motion pattern of the target and to update the motion state through grayscale matching between the predicted target intensity and the observed target intensity. The following section provides a detailed description of this procedure. When estimating the motion pattern of a target using Kalman filtering, the process begins with trajectory initialization. An initial trajectory is established for each candidate target detected in the first frame. Subsequently, the Kalman filtering algorithm is used to predict and estimate the states of candidate targets in subsequent frames. The predicted targets in the new frame are then matched with the existing motion trajectories using the proposed inverse optical flow matching algorithm, thereby updating the motion tracks. If a candidate target in the new frame does not correspond to any existing trajectory, a new trajectory initialization is performed for that candidate target. Conversely, if a trajectory fails to associate with any candidate target for Th consecutive frames, it is removed from further tracking. After completing the data association process in the current frame, the trajectory parameters are updated using the feature information of the associated candidate targets.

Because the time interval between adjacent frames is short, the motion of candidate targets can be approximated as linear motion. Therefore, the system state vector $x_{t}$ at time *t* can be expressed as Equation (25):

(25) $$x_{t}=F_{t}x_{t-1}+B_{t}{\vec{u}}_{t}+w_{t},$$

where $F_{t}$ denotes the state transition matrix, $x_{t-1}$ represents the system state vector at time t−1, and $F_{t}x_{t-1}$ maps the previous candidate target state vector to time *t*. ${\vec{u}}_{t}$ denotes the motion observation vector, and $B_{t}$ is the control input matrix, which maps ${\vec{u}}_{t}$ to the state vector and describes the influence of the control input on the current state. $w_{t}$ represents the process noise, which is assumed to follow a Gaussian distribution with zero mean and covariance matrix $Q_{t}$. The observation $z_{t}$ of the true system state can be expressed as Equation (26):

(26) $$z_{t}=H_{t}x_{t}+v_{t},$$

where $H_{t}$ represents the observation model, which maps the true state space to the observation space. $v_{t}$ denotes the observation noise, which is assumed to follow a zero-mean Gaussian distribution with covariance matrix $R_{t}$.

Based on the above formulation, the trajectory of a candidate target can be predicted and denoted as ${\hat{x}}_{t}^{-}$:

(27) $${\hat{x}}_{t}^{-}=F_{t}{\hat{x}}_{t-1}^{-}+B_{t}u_{t},$$

where ${\hat{x}}_{t}$ represents the predicted state estimate at time *t*. To obtain the optimal estimate, a correction step is performed, yielding ${\hat{x}}_{t}^{-}$. The state transition matrix $F_{t}$ links the system state from the previous time step to the current time step and describes the state evolution from t−1 to *t*. Accordingly, the predicted state of the candidate target at time *t* can be written as Equation (28):

(28) $${\hat{x}}_{t}^{-}=\left[{\hat{x}}^{-}\left(t\right),{\hat{y}}^{-}\left(t\right),{\hat{v}}_{x}^{-}\left(t\right),{\hat{v}}_{y}^{-}\left(t\right)\right],$$

where $\left[{\hat{x}}^{-}\left(t\right),{\hat{y}}^{-}\left(t\right)\right]$ represents the position components of the target at time *t*, and $\left[{\hat{v}}_{x}^{-}\left(t\right),{\hat{v}}_{y}^{-}\left(t\right)\right]$ denotes the velocity components of the centroid of the candidate target along the horizontal and vertical directions, respectively.

As shown in Equation (29), the covariance matrix ${P_{t}}^{-}$ of ${\hat{x}}_{t}^{-}$ represents the uncertainty of the system state estimate at time *t*:

(29) $${P_{t}}^{-}=F_{t}P_{t-1}{F_{t}}^{T}+Q_{t},$$

where $Q_{t}$ denotes the process covariance matrix.

Next, the state estimate is refined through the update step of the Kalman filter:

(30) $${\hat{x}}_{t}={\hat{x}}_{t}^{-}+K_{t}\left(z_{t}-H_{t}{\hat{x}}_{t}^{-}\right),$$

where $K_{t}$ denotes the optimal Kalman gain at time *t*, defined as Equation (31):

(31) $$K_{t}={P_{t}}^{-}{H_{t}}^{T}{\left(H_{t}{P_{t}}^{-}{H_{t}}^{T}+R_{t}\right)}^{-1}.$$

The updated optimal covariance matrix $P_{t}$ of the system state at time *t* is then given as Equation (32):

(32) $$P_{t}=\left(I-K_{t}H_{t}\right){P_{t}}^{-},$$

where *I* represents the identity matrix.

**Figure 15:**
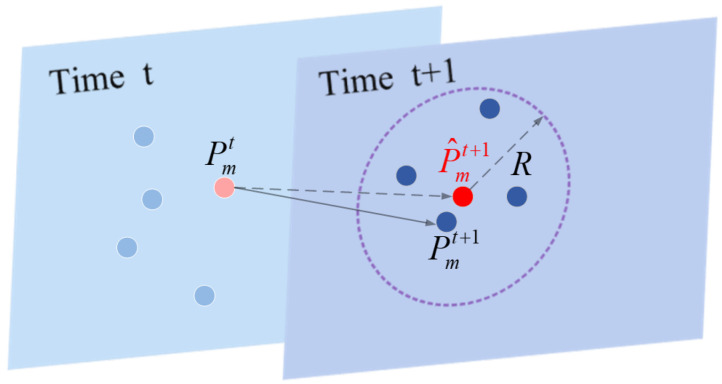
Schematic diagram of candidate target association.

Figure 15 illustrates the candidate trajectory extraction algorithm employed in this study. At time *t*, suppose there are *m* candidate trajectories, each corresponding to a target region $\left\{P_{1}^{t},P_{2}^{t},\ldots ,P_{m}^{t}\right\}$. The objective is to predict the positions of these trajectories at time t+1. Let BB denote the grayscale value of a specific pixel within the target region at time *t*. The goal is to estimate the grayscale value ${\hat{I}}^{t+1}$ of the target at time t+1, which is denoted as $I\left(x+dx,y+dy,t+dt\right)$ in Equation (23).

Taking point $P_{m}$ as an example, its true position at time *t* lies within a pale red circular region centered at $P_{m}^{t}$. By applying Kalman filtering, the target position at time t+1 can be predicted, denoted as ${\hat{P}}_{m}^{t+1}$. According to Equation (23), the brightness values of each pixel in the predicted region are estimated, which are illustrated as the dark red region in Figure 15. Using the centroid of ${\hat{P}}_{m}^{t+1}$ as the origin, a gating window with radius *R* is established. If no candidate targets are found within the gating window, the target is recorded as temporarily lost. When a trajectory fails to associate with any candidate target for Th consecutive frames, it is removed from subsequent association processes. If only a single candidate target appears within the gating window, it is directly selected as the matched target. If multiple candidate targets fall within the gating window, a matching degree is computed, and the region with the highest matching degree is selected for association.

We next analyze the case in which multiple candidate targets exist, as illustrated in Figure 15. Let $\left\{P_{1}^{t+1},P_{2}^{t+1},\ldots ,P_{n}^{t+1}\right\}$ denote the candidate targets within the gating window at time t+1. The matching degree between each of the n candidate targets and the predicted target region ${\hat{P}}_{m}^{t+1}$ is calculated.

In this work, cosine similarity [49] is employed to measure the similarity between two vectors. Its main advantage is that it is insensitive to the magnitude of the vectors and focuses only on their directional consistency. Consequently, even when there are noticeable differences in the absolute pixel intensities between the candidate target and the predicted target, a high similarity score can still be obtained if their directional characteristics are similar. This property is particularly suitable for scenarios in which the grayscale characteristics of a target may vary slightly between frames due to illumination changes or other environmental factors. Accordingly, the matching degree *M* used for candidate target filtering is defined as Equation (33):

(33) $$\begin{array}{cc} M\left({\hat{P}}_{m}^{t+1},P_{i}^{t+1}\right)=\frac{dot\left({\hat{P}}_{m}^{t+1}\left(:\right),P_{i}^{t+1}\left(:\right)\right)}{\left|{\hat{P}}_{m}^{t+1}\left(:\right)\right|\cdot \left|P_{i}^{t+1}\left(:\right)\right|}, & i=1,\ldots ,n \end{array},$$

where $dot\left(\cdot \right)$ denotes the vector dot product, $\left|\cdot \right|$ represents the vector norm, and ${\hat{P}}_{m}^{t+1}\left(:\right)$ is the vector obtained by transforming the predicted candidate target region matrix into a column vector.

The value of the matching degree *M* lies within $\left[0,1\right]$. A value closer to 1 indicates higher similarity between the two regions, whereas a value closer to 0 indicates weak correlation, implying that the vectors are approximately orthogonal. Therefore, it is sufficient to compute the matching degrees between the candidate targets within the gating window and the predicted target region, and select the candidate with the highest matching degree as the associated trajectory object. If multiple candidates share the same maximum matching degree, the one closest to the predicted position is chosen.

The above procedure is repeated iteratively until all frames in the image sequence have been processed, thereby extracting all candidate trajectories. The pseudo-code of the candidate trajectory extraction algorithm based on inverse optical flow matching proposed in this paper is presented in Algorithm A4.

### 3.4. Multi-Feature Fusion Trajectory Filtering Strategy

The candidate trajectory set typically contains both true targets and false detections. To reduce the false alarm rate, improve trajectory quality, and conserve computational resources, this study manages and filters candidate trajectories by estimating their confidence based on multiple discriminative features. At this stage, the dominant interference arises from sea clutter, which often exhibits visual characteristics similar to those of small ship targets and cannot be effectively suppressed using single-frame image features alone. Therefore, a multi-feature fusion trajectory filtering strategy is developed by exploiting the statistical differences between real ship targets and sea clutter.

**Figure 16:**
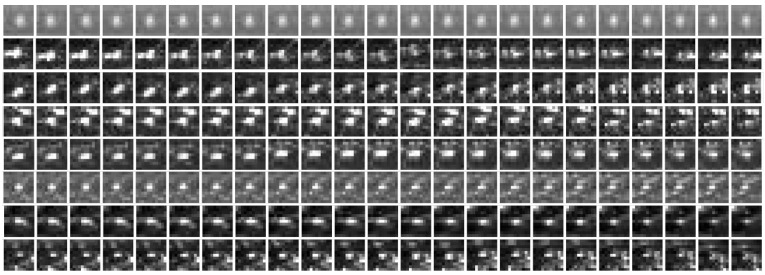
Candidate target regions extracted from consecutive frames.Candidate target regions extracted from 50 consecutive frames for one confirmed ship target (**top row**) and representative sea-clutter trajectories (**others**).

Figure 16 presents several candidate target regions extracted from consecutive frames, including both real ship targets and sea clutter. These two types of regions exhibit noticeable differences in several characteristics, including motion entropy, peak signal-to-noise ratio, and trajectory lifecycle.

Motion entropy (ME) is used to characterize the motion information within image sequences by quantifying the pixel variation within candidate target regions. It reflects the degree of motion randomness in the region and is defined as Equation (34):

(34) $$ME=-\sum_{i=1}^{N}p_{i}\log_{2}\left(p_{i}\right),$$

where $p_{i}$ denotes the motion probability of the i-th pixel location within the candidate target region.

**Figure 17:**
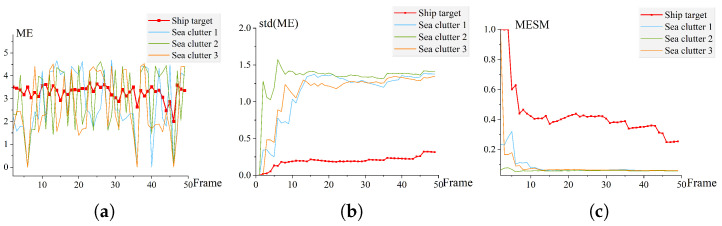
Motion entropy analysis of candidate trajectory. (**a**) Variation in motion entropy. (**b**) Variation in the standard deviation of motion entropy. (**c**) Variation in motion entropy stability measure.

Figure 17a illustrates the variation in motion entropy for one set of ship targets and three sets of sea clutter over 50 consecutive frames. Compared with sea clutter, ship targets generally maintain a more stable appearance, resulting in smaller variations in motion entropy between adjacent frames. As shown in Figure 17b, the standard deviation of motion entropy for ship targets is significantly smaller than that of sea clutter. Motion Entropy Stability Measure (MESM) is introduced and defined as Equation (35) to quantitatively measure this stability:

(35) $$MESM=\min\left[\frac{1}{std\left(ME\right)},1\right],$$

where $std\left(ME\right)$ denotes the standard deviation of motion entropy over the trajectory. Figure 17c shows the MESM curves for ship targets and sea clutter, indicating that ship targets exhibit significantly higher stability values.

The Peak Signal-to-Noise Ratio (PSNR) measures the ratio between the maximum possible pixel value and the mean squared error introduced by variations between image regions. It can therefore indirectly reflect the visual stability of candidate target trajectories. PSNR is calculated as Equation (36):

(36) $$PSNR=10\log_{10}\left(\frac{MAX^{2}}{MSE}\right),$$

where MAX denotes the maximum possible pixel value. For the 8-bit images used in this study, MAX=255. The Mean Squared Error (MSE) is the mean square error, calculated as the mean of the squares of the differences between corresponding pixels in candidate target regions before and after the trajectory and defined as Equation (37):

(37) $$MSE=\frac{1}{mn}\sum_{i=1}^{m}\sum_{j=1}^{n}{\left[I_{1}\left(i,j\right)-I_{2}\left(i,j\right)\right]}^{2},$$

where $I_{1}\left(i,j\right)$ and $I_{2}\left(i,j\right)$ represent the pixel intensities at the i-th row and j-th column of the candidate target regions before and after trajectory evolution, respectively, and m×n denotes the size of the target region.

**Figure 18:**
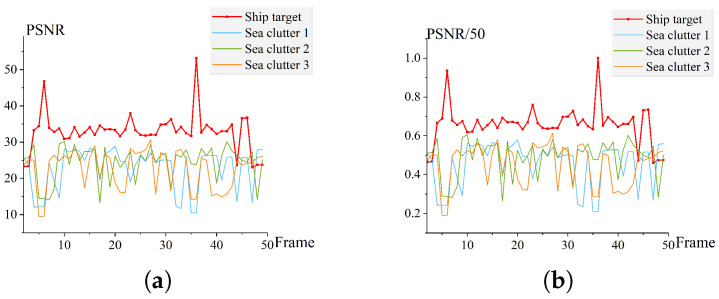
Peak signal-to-noise ratio analysis of candidate trajectory. (**a**) Variation in peak signal-to-noise ratio. (**b**) Variation in normalized peak signal-to-noise ratio.

Figure 18a shows the PSNR variations for one set of ship targets and three sets of sea clutter over 50 consecutive frames. Compared with sea clutter, ship targets exhibit higher PSNR values with smaller fluctuations over short time intervals, indicating greater visual stability. In contrast, sea clutter produces lower and more unstable PSNR values. Therefore, PSNR can serve as an effective criterion for distinguishing ship targets from sea clutter.

For the subsequent confidence calculation, a normalization factor is introduced to scale the PSNR values:

(38) $$PSNR=\min\left(\frac{PSNR}{A},1\right),$$

where *A* is a scaling factor, empirically set to 50. The normalized results are illustrated in Figure 18b.

**Figure 19:**
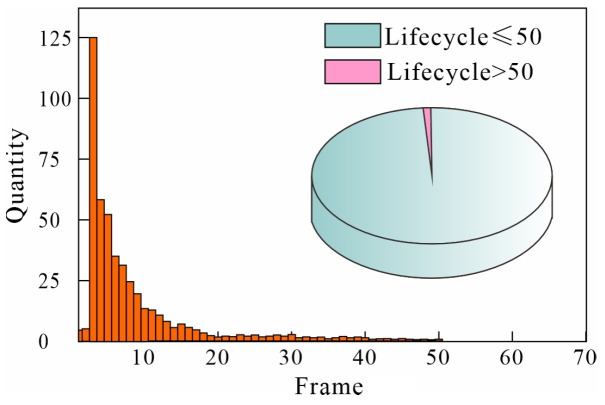
Lifecycle analysis of sea clutter trajectories.

The Lifecycle of sea clutter trajectories in the experimental sequences were statistically analyzed, as shown in Figure 19. The results indicate that approximately 99% of sea clutter trajectories persist for fewer than 50 frames, demonstrating that sea clutter typically exhibits short temporal persistence.

To ensure balanced feature weighting, the trajectory lifecycle is normalized to the range $\left[0,50\right]$, as Equation (39):

(39) $$Lifecycle=\min\left(\frac{Lifecycle}{A},1\right),$$

where Lifecycle denotes the trajectory lifecycle and *A* is an empirical scaling factor set to 50, which is chosen based on the statistical finding that 99% of sea clutter trajectories persist for fewer than 50 frames (Figure 19).

Based on the above features, a multi-feature fusion confidence metric is defined as

(40) $$C=\omega_{1}MESM+\omega_{2}PSNR+\omega_{3}Lifecycle,$$

where $\omega_{1}$, $\omega_{2}$, and $\omega_{3}$ denote the weights assigned to motion entropy stability, peak signal-to-noise ratio, and lifecycle, respectively. Based on development experience, it is recommended to set 0.3, 0.3 and 0.03, respectively. MESM, PSNR, and Lifecycle represent the normalized values of MESM, PSNR, and trajectory lifecycle.

Candidate trajectories are then evaluated according to their confidence values:

(1) If $C<Th_{1}$, the trajectory is discarded.
(2) If $Th_{1}\leq C\leq Th_{2}$, the trajectory continues to be updated.
(3) If $C>Th_{2}$, the trajectory is confirmed and output as a detected target.

Based on empirical observations in practical applications, the thresholds $Th_{1}$ and $Th_{2}$ are selected accordingly to balance detection sensitivity and false alarm suppression, which set as 0.3 and 0.9.

By integrating multiple complementary features—including motion entropy stability, peak signal-to-noise ratio, and trajectory lifespan—the proposed multi-feature fusion trajectory filtering strategy effectively distinguishes real ship targets from sea clutter, thereby improving the robustness and reliability of the overall detection framework.

## 4. Experimental Results and Analysis

This section verifies the performance of the proposed algorithm through experiments. The experimental test content includes: (1) Analysis of the detection results of the proposed algorithm in this chapter, which presents and analyzes the saliency region extraction effect, DHOG candidate region extraction effect, and the fused results; (2) Real-time performance test, which verifies the real-time applicability of the proposed algorithm by testing the processing speed; (3) Ablation experiment, which quantitatively verifies the individual contribution of each core module of the proposed method to the final detection performance; (4) Comparative analysis of single-frame detection results, where 6 single-frame target detection algorithms are selected as comparison algorithms to test the performance of single-frame detection results; (5) Comparative analysis of multi-frame detection results, where 5 multi-frame target detection algorithms are selected as comparison algorithms to test the performance of multi-frame detection results. All experiments in this chapter are conducted on a desktop computer with 16 GB of memory, an Intel i7-6700 CPU with a main frequency of 3.4 GHz, and the code of all algorithms is implemented in MATLAB R2016a.

To ensure a fair and rigorous comparison, all comparison algorithms (including single-frame methods and multi-frame methods) were implemented strictly in accordance with the parameter settings recommended in their original publications, without any additional parameter tuning or adjustment for the dataset used in this study. The preprocessing and post-processing conditions for all comparison algorithms were also consistent with those described in their original papers, including image normalization, noise reduction, and candidate target filtering criteria, to eliminate the impact of inconsistent implementation conditions on the comparison results. The code sources of the comparison algorithms are either publicly available (with specific links provided) or implemented based on the detailed descriptions in their original publications, ensuring reproducibility.

### 4.1. Experimental Data and Evaluation Metrics

#### 4.1.1. Experimental Data

**Figure 20:**
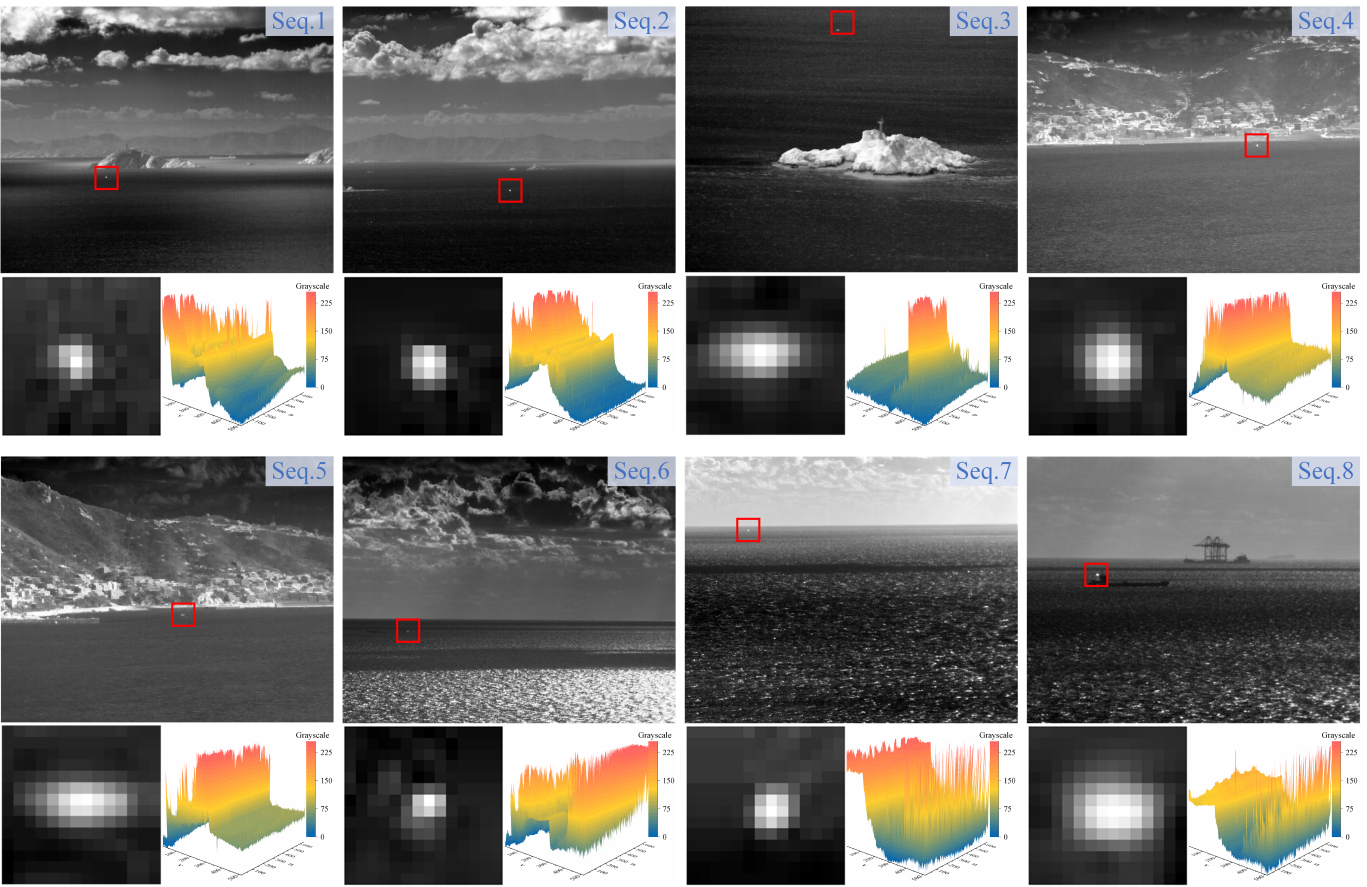
First frames of the experimental sequences, along with local magnified views of the targets and their corresponding 3-D grayscale representations.

This section evaluates the proposed method using eight infrared image sequences containing small targets in sea–sky background environments. The first frame of each sequence is shown in Figure 20, where the target regions are indicated by red bounding boxes. Local magnified views of the targets and their corresponding 3-D grayscale representations are displayed below each image. All targets correspond to maritime vessels or their navigation lights. The sequences were acquired using a cooled infrared camera, and the main specifications of the imaging system are summarized in Table 2.

The dataset covers a variety of typical interference backgrounds encountered in maritime infrared target detection. Specifically, cloud backgrounds constitute the dominant interference in Sequences 1, 2, and 6. Island and coastal backgrounds are the main interference in Sequences 3, 4, and 5. Surface reflections and sea clutter are predominant in Sequences 6, 7, and 8. Detailed information regarding each experimental sequence is summarized in Table 3. The camera frame rate for all sequences is fixed at 50 Hz (see Table 2). The “Total Frames” column indicates the total number of frames used in each experimental sequence.

#### 4.1.2. Evaluation Metrics

To enable quantitative experimental analysis and support subsequent discussion, the evaluation metrics adopted in this study are defined as follows.

(1) Background Suppression Factor

The Background Suppression Factor (BSF) [50] measures the effectiveness of background suppression achieved by a detection algorithm. It is defined as Equation (41):

(41) $$BSF=\frac{\sigma_{in}}{\sigma_{out}},$$

where $\sigma_{in}$ and $\sigma_{out}$ denote the standard deviations of the input image and the processed image, respectively. A larger BSF value indicates stronger suppression of background fluctuations.

(2) Signal-to-Clutter Ratio Gain

The Signal-to-Clutter Ratio Gain (SCRG) [50] evaluates the improvement in target contrast relative to background clutter. It is defined as Equation (42):

(42) $$SCRG=\frac{SCR_{out}}{SCR_{in}},$$

where $\mathrm{SC}R_{in}$ and $\mathrm{SC}R_{out}$ represent the signal-to-clutter ratios of the input and output images, respectively. A higher SCRG indicates that the algorithm enhances the target more effectively while suppressing clutter. The Signal-to-Clutter Ratio (SCR) [50] is defined as Equation (43):

(43) $$SCR=\frac{I_{T}-\mu_{B}}{\sigma_{B}},$$

where $I_{T}$ denotes the mean intensity of the target region, and $\mu_{B}$ and $\sigma_{B}$ denote the mean and standard deviation of the background within a local neighborhood (excluding target pixels), respectively. In this study, all non-target components are treated as clutter. The SCR reflects the relative contrast between the target and its surrounding background, thereby indicating the level of detection difficulty.

(3) Receiver Operating Characteristic Curve

The Receiver Operating Characteristic (ROC) curve [51] characterizes the trade-off between detection performance and false alarms by plotting the detection rate against the false alarm rate.

Here, the Detection Rate (DR) is defined as Equation (44):

(44) $$DR=\frac{N_{dp}}{N_{tp}},$$

where $N_{dp}$ is the number of correctly detected target pixels, and $N_{tp}$ is the total number of ground-truth target pixels.

The False Alarm Rate (FAR) is defined as Equation (45):

(45) $$FAR=\frac{N_{f}}{N},$$

where $N_{f}$ denotes the number of falsely detected pixels, and *N* is the total number of pixels in the image.

### 4.2. Experiments and Discussion

#### 4.2.1. Detection Process and Results of the Proposed Method

**Figure 21:**
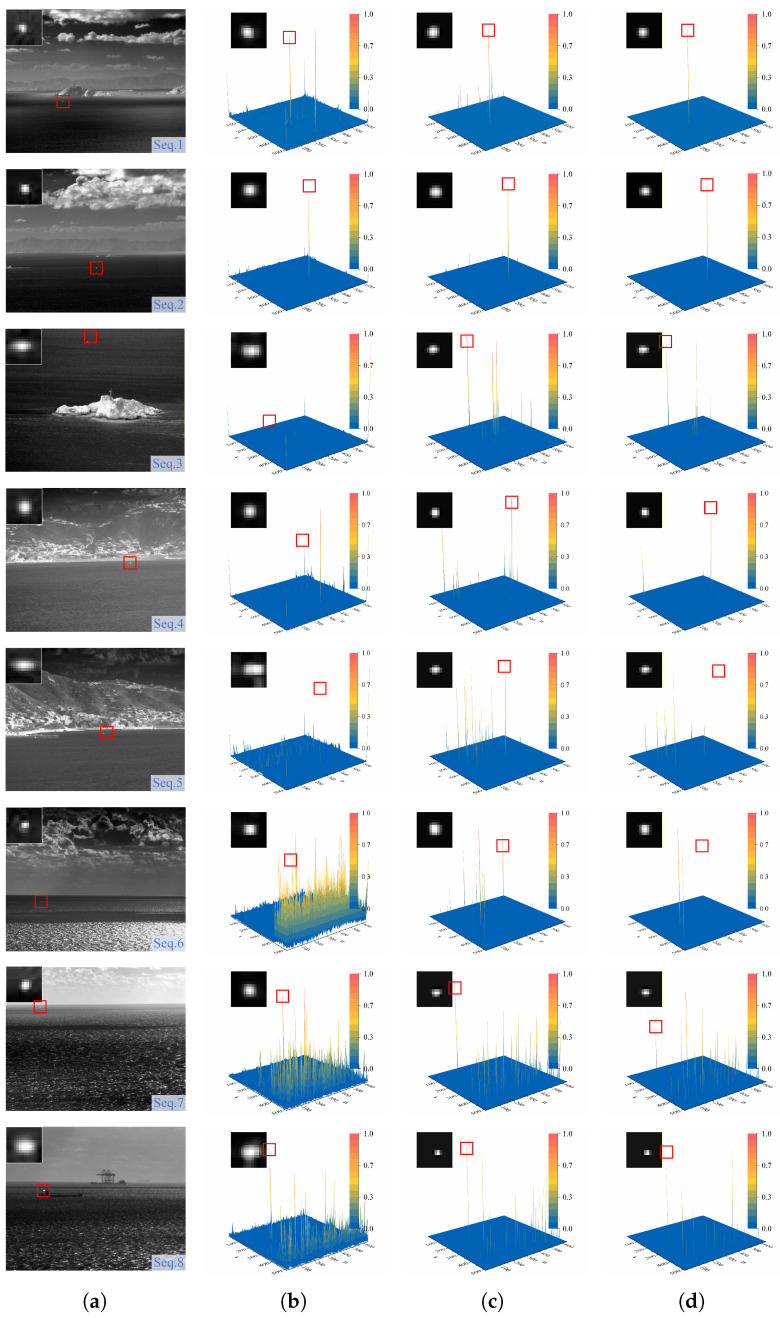
Experimental results of key steps. (**a**) Original images of 8 image sequences. (**b**) Saliency maps of 8 image sequences. (**c**) DHOG responses of 8 image sequences. (**d**) SH fusion maps of 8 image sequences.

To qualitatively demonstrate the effectiveness of the proposed approach, experiments were conducted on the eight ship target sequences described in Section 4.1. The intermediate processing results of the key stages are illustrated in Figure 21, where magnified views of the target regions are shown in the upper-left corner of each corresponding image.

After the 8-bit raw infrared image is input, as shown in Figure 21a, a saliency map is first generated using the log-spectrum-based saliency extraction method. As illustrated in Figure 21b, high-frequency components such as targets, cloud edges, island boundaries, and sea clutter are prominently highlighted. Next, candidate regions are extracted based on HOG differences, producing the DHOG response map, as shown in Figure 21c. In this stage, potential targets, sea clutter, and some edge structures in cloud or island regions are enhanced. Finally, the saliency map and DHOG map are fused to obtain the SH fusion map, shown in Figure 21d. The results indicate that the target response is significantly strengthened, while most background regions are effectively suppressed, except for certain strong sea clutter structures. Since strong sea clutter may exhibit morphological characteristics similar to those of true targets, it cannot be completely eliminated using grayscale features alone. Therefore, further false alarm suppression through temporal feature analysis is required.

In the following subsections, the proposed inter-frame trajectory-based detection algorithm will be quantitatively compared with several representative methods.

#### 4.2.2. Real-Time Performance Test

To verify the real-time applicability of the proposed method, a real-time performance test is conducted under the same hardware and software environment as the aforementioned experiments. The test uses 640 × 512 pixel images as input, and records the average processing time per frame (ms/frame). In addition, the computational time of each core module of the proposed method is decomposed to further illustrate the lightweight characteristics of the framework. Each algorithm is tested 100 times continuously, and the average value is taken to eliminate the impact of accidental factors on the test results.

To further illustrate the lightweight characteristics of the proposed method, the computational time of each core module is decomposed and analyzed under the same test environment. The results show that the multi-scale DHOG candidate extraction module takes an average of 18.7 ms per frame, the saliency fusion module takes 4.2 ms per frame, the inverse optical flow-based trajectory extraction module takes 11.3 ms per frame, and the multi-feature trajectory filtering module takes 4.4 ms per frame. The total average processing time per frame is 38.6 ms, which is lower than most baseline algorithms. The low computational complexity is mainly attributed to the local gradient computation (avoiding global feature extraction) and lightweight trajectory filtering (simplifying complex motion modeling), which is consistent with the description in the conclusion. The frame rate of the proposed method reaches 25.9 FPS, which is higher than the 25 FPS required for real-time maritime surveillance, fully verifying its suitability for real-time application scenarios.

#### 4.2.3. Ablation Experiment

To quantitatively verify the individual contribution of each core module of the proposed method (saliency map, multi-scale DHOG, inverse optical flow matching, and multi-feature trajectory filtering) and respond to the concern about module contribution, ablation experiments are conducted under the same hardware and software environment as the aforementioned experiments. The ablation experiments adopt a step-by-step configuration strategy, gradually adding each core module to form different experimental groups, and compare their detection performance to clarify the performance improvement brought by each module. The experimental groups are set as follows: (1) Saliency only: Only the log-spectrum-based saliency map module is used for target detection; (2) DHOG only: Only the multi-scale DHOG candidate extraction module is used for target detection; (3) Saliency + DHOG: The saliency map module is fused with the multi-scale DHOG module; (4) Saliency + DHOG + Inverse optical flow: On the basis of the third group, the inverse optical flow-based trajectory extraction module is added; (5) Full model (Proposed Method): All four core modules are integrated (saliency map + multi-scale DHOG + inverse optical flow + multi-feature trajectory filtering). The performance indicators used for comparison include BSF (Background Suppression Factor), SCRG (Signal-to-Clutter Ratio Gain), and AUC (Area Under the Receiver Operating Characteristic Curve), which are consistent with the indicators used in the previous comparative experiments. Each experimental group is tested on all 8 infrared maritime sequences, and the average value of each performance indicator is calculated to ensure the reliability of the experimental results.

The results of the ablation experiment (Table 4) clearly quantify the individual contribution of each core module to the final detection performance of the proposed method. Specifically, the analysis is as follows: (1) The “Saliency only” group achieves relatively low performance (AUC = 0.821), indicating that the saliency map module alone can only initially enhance the target contrast but cannot effectively suppress complex background clutter. (2) The “DHOG only” group (AUC = 0.857) performs better than the “Saliency only” group, which verifies that the multi-scale DHOG module has a stronger ability to extract target gradient features and suppress background edges. (3) The “Saliency + DHOG” group (AUC = 0.915) shows a significant performance improvement compared with the single-module groups, indicating that the fusion of saliency map and multi-scale DHOG can complement each other, effectively enhancing target responses and suppressing background clutter. (4) After adding the inverse optical flow-based trajectory extraction module (“Saliency + DHOG + Inverse optical flow”), the AUC increases to 0.942, which proves that the temporal consistency exploited by the inverse optical flow module can effectively filter out false alarms caused by static background interference. (5) The full model, which integrates all four core modules, achieves the best performance (AUC = 0.963), and the multi-feature trajectory filtering module further improves the detection reliability by integrating motion entropy stability, peak signal-to-noise ratio, and trajectory lifespan, reducing the false alarm rate. In summary, each core module of the proposed method contributes positively to the detection performance, and the step-by-step fusion of modules forms a coherent framework that significantly improves the target–background separability and detection robustness.

#### 4.2.4. Comparative Analysis of Single-Frame Detection Results

In the candidate target extraction stage, this paper proposes a HOG-difference-based candidate region extraction algorithm, which is further fused with a log-spectrum saliency map to improve detection performance.

To evaluate the effectiveness of the proposed method, six representative single-frame infrared small-target detection algorithms were selected for comparison: LCM [11], MPCM [52], MLHM [53], LoSH^3^ [54], as well as ADMD [55] and LogTFNN [56]. Among these methods, LCM performs target detection through local contrast measurement. MPCM enhances targets using multi-scale patch contrast measures. MLHM improves detection performance through multi-scale local homogeneity measurements. LoSH^3^ is based on the matched-filter signal-to-clutter ratio enhancement mechanism, constructing a small-target model by analyzing the point spread function (PSF). ADMD proposes an absolute directional mean difference operator for target enhancement. LogTFNN extends tensor rank to tensor fiber rank, using a log-based tensor fiber kernel norm for nonconvex rank approximation and employing hyper-total variation regularization to constrain the background.

**Figure 22:**
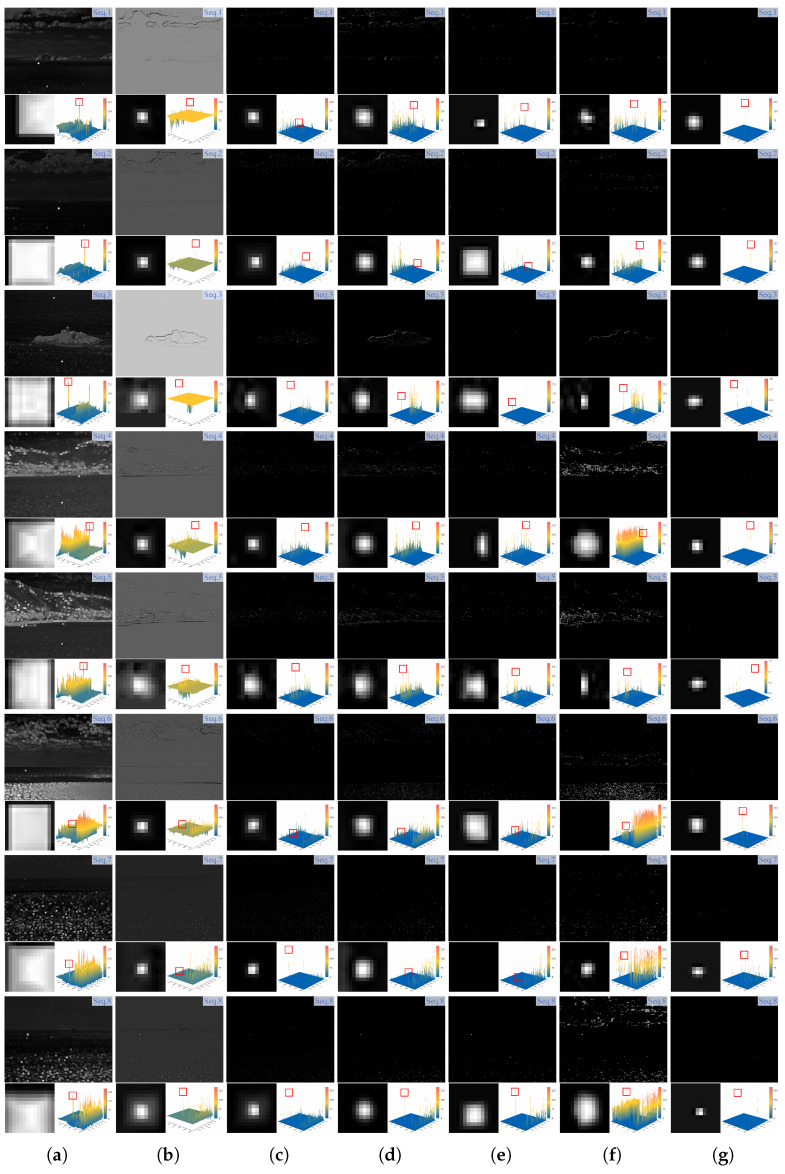
Comparison of results between comparable methods and our method. (**a**) Results of the LCM for 8 image sequences. (**b**) Results of the MPCM for 8 image sequences. (**c**) Results of the MLHM for 8 image sequences. (**d**) Results of the LoSH^3^ for 8 image sequences. (**e**) Results of the ADMD for 8 image sequences. (**f**) Results of the LogTFNN for 8 image sequences. (**g**) Results of the our method for 8 image sequences.

A qualitative comparison was first conducted by analyzing the filtered output images produced by each algorithm. Figure 22 presents the filtering results and their corresponding 3-D intensity maps, along with magnified views of the target regions.

The results indicate that LCM and MPCM effectively enhance targets in relatively clean backgrounds. However, their ability to suppress complex backgrounds—such as the mountainous terrain in Sequence 5 and strong sea clutter in Sequences 7 and 8—is limited. This limitation increases the difficulty of subsequent candidate screening and leads to a higher false alarm rate. The MLHM method demonstrates strong background suppression capability; however, the resulting target peaks are relatively weak, which complicates threshold-based target extraction. For LoSH^3^, ADMD, and LogTFNN, the contrast between target peaks and background responses is relatively small. As a result, these methods may either miss true targets or produce an excessive number of candidate regions. In contrast, the proposed method consistently produces stronger target responses while maintaining lower residual background clutter across all sequences. This property not only improves detection reliability but also simplifies the subsequent trajectory association and filtering stages, making it particularly suitable for candidate target extraction.

To quantitatively evaluate detection performance, two commonly used metrics were adopted: Background Suppression Factor (BSF) and Signal-to-Clutter Ratio Gain (SCRG). In the result tables, the best performance for each sequence is highlighted in bold red, while the second-best result is marked in bold blue.

The BSF comparison results are presented in Table 5, and the SCRG results are shown in Table 6.

From Table 5, it can be observed that the proposed method achieves the highest BSF values in most sequences, outperforming the second-best algorithm by at least 15.76%, which demonstrates its superior background suppression capability.

Similarly, Table 6 shows that the proposed method achieves the highest SCRG values in most sequences, except for Sequence 7. These results indicate that the proposed algorithm provides stronger target enhancement relative to background clutter.

The Receiver Operating Characteristic (ROC) curve illustrates the relationship between the Detection Rate (DR) and the False Alarm Rate (FAR). In general, a curve closer to the upper-left corner represents better detection performance, as it indicates a higher detection probability at a given false alarm rate.

**Figure 23:**
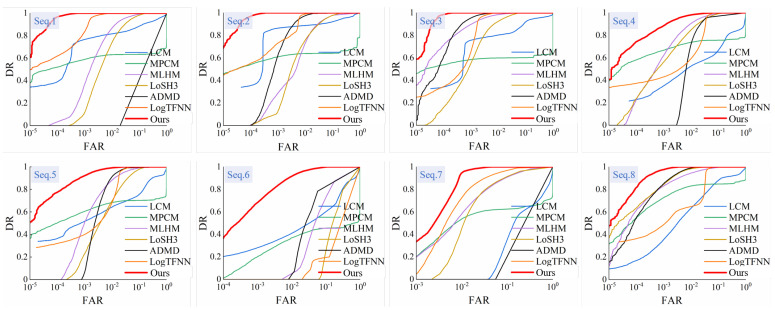
ROC curves for single-frame target detection between the comparison methods and our method.

Figure 23 presents the ROC curves of all compared methods and the proposed algorithm for single-frame detection across the eight sequences. The comparison reveals the following observations: LoSH^3^ and ADMD exhibit the weakest performance, with their ROC curves consistently positioned lower than those of the other methods. MLHM shows relatively stable performance but remains inferior to the proposed approach. LCM, MPCM, and LogTFNN demonstrate inconsistent performance across different datasets.

In contrast, the proposed method consistently achieves higher ROC curves across all sequences, indicating that it can attain a higher detection rate under the same false alarm rate. This advantage provides a stronger foundation for subsequent trajectory association and multi-frame filtering.

#### 4.2.5. Comparative Analysis of Multi-Frame Detection Results

This paper proposes a candidate trajectory extraction algorithm based on inverse optical flow matching, combined with a multi-feature trajectory filtering strategy to achieve reliable detection of small ship targets. To evaluate the effectiveness of the proposed method, comparative experiments were conducted using five representative multi-frame infrared small-target detection algorithms: STLCF [57], MSL-STIPT [58], 4DTT [59], 4DTR [59], and ECA-STT [60].

Among these methods, STLCF is a spatiotemporal local contrast filter designed based on spatial and temporal local contrast measures. MSL-STIPT employs multi-subspace learning and a spatiotemporal tensor representation, enabling accurate recovery of target components through an efficient optimization framework based on the alternating direction method of multipliers (ADMM). 4DTT and 4DTR construct a four-dimensional infrared tensor from image sequences and perform tensor decomposition using tensor train (TT) and tensor ring (TR) representations, respectively. ECA-STT introduces an edge- and corner-aware spatiotemporal tensor model to enhance detection robustness.

**Figure 24:**
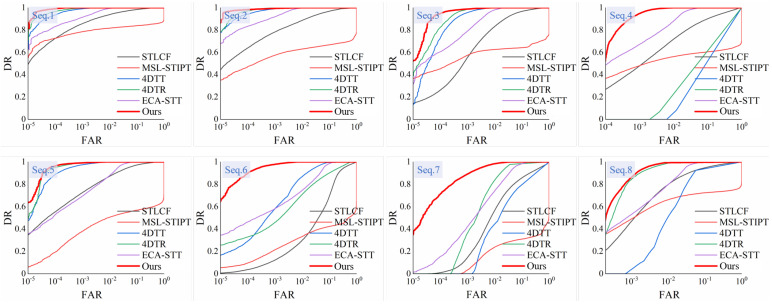
ROC curves for multi-frame target detection between the comparison methods and our method.

In the experiments, the ROC curve is used as the primary evaluation metric to assess the overall detection performance of different algorithms. The ROC curves illustrate the relationship between the DR and the FAR. The comparison results are shown in Figure 24.

From the experimental results, several observations can be made. STLCF and MSL-STIPT exhibit the weakest detection performance, as their ROC curves remain consistently lower than those of the other methods across most sequences. Although 4DTT and 4DTR achieve relatively good results, their performance is still inferior to that of the proposed method. Meanwhile, ECA-STT performs well on certain sequences but shows limited stability across different datasets.

In contrast, the proposed algorithm consistently achieves higher ROC curves across all sequences, indicating that it provides a higher detection rate under the same false alarm rate. Combined with the previously presented single-frame detection results and trajectory-based multi-frame analysis, these findings demonstrate that the proposed method offers superior overall detection performance compared with existing approaches.

## 5. Conclusions

Detecting small ship targets in sea–sky backgrounds remains challenging due to complex interference from clouds, islands, and sea clutter, as well as the loss of target detail under long-range imaging conditions. To address these issues, this paper proposes a robust spatiotemporal detection framework tailored for multi-scale bright and dark targets with varying shapes. First, a multi-scale HOG-difference-based candidate extraction method is developed by analyzing the gradient distribution differences between targets and background regions. By integrating this operator with saliency detection, the method effectively enhances targets while suppressing background clutter. Subsequently, an inverse optical flow-based trajectory extraction algorithm is introduced to exploit temporal consistency across image sequences. To further improve detection reliability, a multi-feature trajectory filtering strategy is designed, incorporating motion entropy stability, peak signal-to-noise ratio, and trajectory lifespan. This enables effective discrimination between true targets and background clutter. Experimental results demonstrate that the proposed method achieves a higher detection rate under the same false alarm rate compared with existing approaches.

From a theoretical perspective, the framework unifies spectral saliency, multi-scale gradient modeling, and temporal consistency into a coherent spatiotemporal mechanism that enhances target–background separability, which fills the gap related to the insufficient integration of spatial gradient features and temporal trajectory information in existing small ship detection methods. From an engineering perspective, the reliance on local gradient computation and lightweight trajectory filtering ensures relatively low computational complexity, making the method suitable for real-time maritime surveillance applications—this provides a practical technical solution for low-cost, real-time maritime target monitoring systems, which is of great significance for improving the efficiency of maritime safety supervision and reducing manual monitoring costs.

Limitations and Future Work: Despite the promising performance of the proposed method, several limitations should be acknowledged. First, the experimental validation is based on eight self-collected sequences, and the dataset size is relatively small. Second, some parameters are selected empirically, which may reduce the adaptability of the method to different sea conditions. Third, the confidence equation has not been fully verified under extremely low-signal-to-noise ratio (SNR) conditions, and its robustness needs further improvement.

To address these limitations, future research directions are proposed as follows: First, expand the dataset size by collecting more diverse maritime sequences (including different sea conditions, weather, and target types) and strive to release a public dataset to promote related research. Second, optimize the parameter selection mechanism by introducing adaptive parameter tuning methods (e.g., intelligent optimization algorithms) to improve the method’s adaptability. Third, refine the confidence equation to enhance its robustness under extremely low-SNR conditions.

## Figures and Tables

**Table 1:** Estimated parameters of Gaussian model coefficients.

Image	γ	$\sigma_{x}$	$\sigma_{y}$
Image 1	85.2587	6.8341	1.3352
Image 2	94.5214	9.1207	2.0859
Image 3	91.8418	4.6712	2.6751
Image 4	100.2547	4.3391	2.5206
Image 5	135.8413	6.1673	2.6707
Image 6	88.0976	2.3313	2.3364

**Table 2:** The main specifications of the imaging system.

Indicator	Parameters
Working band (μm)	3∼5
Focal length (mm)	100
Frame rate (Hz)	50
Image resolution (pixel)	640×512
Pixel size (μm)	15×15
Field of view angle (°)	5.5×4.4

**Table 3:** Details of experimental sequences.

Sequence	Total Frames	Resolution (Pixel)	Target Type	Target Size (Pixel)	Average Speed of Target (Pixel/Frame)
Seq.1	200	640×512	Ship	3×4	0.05
Seq.2	100	640×512	Ship	4×4	0.12
Seq.3	200	640×512	Ship	10×5	0.07
Seq.4	200	640×512	Ship	5×5	0.10
Seq.5	100	640×512	Ship	12×5	0.15
Seq.6	200	640×512	Navigation light	3×3	0.06
Seq.7	200	640×512	Ship	3×4	0.09
Seq.8	200	640×512	Navigation light	6×6	0.13

**Table 4:** Ablation experiment results.

Experimental Configuration (Module Combination)	$\bar{\mathrm{BSF}}$	$\bar{\mathrm{SCRG}}$	$\bar{\mathrm{AUC}}$
Saliency only	12.8	$8.3\times 10^{3}$	0.821
DHOG only	15.6	$10.5\times 10^{3}$	0.857
Saliency + DHOG	21.3	$13.8\times 10^{3}$	0.915
Saliency + DHOG + Inverse optical flow	34.5	$15.1\times 10^{3}$	0.942
Full model (Proposed Method)	45.2	$22.3\times 10^{3}$	0.963

**Table 5:** The BSF between the comparison methods and our method. The results marked in red denote the best performance for each sequence, while the results marked in blue represent the second-best.

Sequence	LCM	MPCM	MLHM	LoSH^3^	ADMD	LogTPNN	Ours
Sq.1	3.2144	7.1654	14.6686	6.7822	16.0718	14.9353	52.9977
Sq.2	5.4592	12.5593	24.7023	11.1594	25.1473	9.2456	64.5205
Sq.3	2.5365	8.0551	18.0473	7.3156	32.9232	6.6498	38.1129
Sq.4	1.2572	8.2552	11.9247	5.7062	10.1279	1.5500	46.4737
Sq.5	1.3259	6.7992	10.8234	5.1706	11.6332	2.1534	32.3002
Sq.6	1.3049	8.5923	20.5002	6.3510	13.6476	2.0312	44.6342
Sq.7	3.4943	19.1276	56.9076	24.8068	25.7719	5.8091	37.9847
Sq.8	2.5232	12.7605	15.3809	13.2594	14.4251	2.0582	44.2541

**Table 6:** The SCRG between the comparison methods and our method. The results marked in red denote the best performance for each sequence, while the results marked in blue represent the second-best.

Sequence	LCM	MPCM	MLHM	LoSH^3^	ADMD	LogTPNN	Ours
Sq.1	4.10	8.39	2333.50	1216.70	88.43	10,816.00	26,507.89
Sq.2	1.16	2.32	6.69	7.23	1.18	7.74	3186.20
Sq.3	1.93	5.08	19.01	5.62	3.23	7.95	4871.37
Sq.4	1.43	5.50	21,397.39	7.47	891.79	567.14	51,800.52
Sq.5	0.83	2.45	1787.50	2.54	326.50	1454.75	8604.77
Sq.6	1.72	1.59	16.14	35.49	43.28	630.67	9934.90
Sq.7	2.12	2.98	3509.90	3.74	0.01	64,723.16	54,064.31
Sq.8	3.45	4.55	4.55	14.52	167.56	2538.80	19,575.08

## Data Availability

The dataset is available on request from the authors.

## Appendix A. Pseudo-Code of Proposed Algorithms

The pseudocodes for the core algorithms of the proposed method are presented in this appendix, including the single-scale DHOG map acquisition, multi-scale DHOG map acquisition, candidate target extraction, and inverse optical flow-based candidate trajectory extraction. These algorithms detail the step-by-step implementation logic of key modules in the spatial feature enhancement and temporal trajectory analysis stages, ensuring the reproducibility of the method. The corresponding notations and parameter settings are consistent with those described in the main text.

**Algorithm A1** Single-scale DHOG map acquisition
**Input:** Infrared image **Output:** Single-scale DHOG map **Do:** 1: Compute image gradients according to Equations (2) and (3). 2: Filter the entire image using a sliding window, where each window contains 9 cells. 3: Compute the HOG descriptor for each cell within the sliding window. 4: Calculate the HOG difference values of the 8 regions according to Equation (10). 5: Compute the DHOG value at each sliding window position according to Equation (11), until the window traverses the entire infrared image. 6: Obtain the single-scale DHOG map.

**Algorithm A2** Multi-scale DHOG map acquisition
**Input:** Infrared image **Output:** Multi-scale DHOG map **Do:** 1: Create sliding windows composed of cells of different sizes. 2: **for** l=1 **to** $l_{\max}$ **do** 3: Obtain the single-scale DHOG map $H_{d}^{l}$ according to Algorithm A1. 4: **end for** 5: **for** i=1 **to** *m* **do** 6: **for** j=1 **to** *n* **do** 7: $\hat{H}\left(i,j\right)=\max_{l=1,2,\ldots ,l_{\max}}H_{d}^{l}\left(i,j\right)$ 8: **end for** 9: **end for** 10: Obtain the multi-scale DHOG map.

**Algorithm A3** Computing fused feature map and extracting candidate targets
**Input:** Saliency map, multi-scale DHOG map **Output:** Centroid coordinates of candidate targets **Do:** 1: **for** i=1 **to** *m* **do** 2: **for** j=1 **to** *n* **do** 3: $SH\left(i,j\right)=S\left(i,j\right)\times \hat{H}\left(i,j\right)$ 4: **end for** 5: **end for** 6: Compute the threshold Th according to Equation (16). 7: Obtain the binary segmentation result based on the threshold Th. 8: Obtain the centroid coordinates of each candidate target according to Equation (17).

**Algorithm A4** Candidate trajectory extraction algorithm based on inverse optical flow matching
**Input:** Centroid coordinates of candidate target **Output:** Updated trajectories of candidate targets **Do:** 1: Initialize trajectories for each new candidate target in the frame, and update the number of candidate targets *n*. 2: **for** i=1 **to** *n* **do** 3: Estimate the state vector ${\hat{x}}_{i}^{-}$ at the next time step according to Equation (27). 4: Compute the covariance matrix of $P_{i}^{-}$ according to Equation (29). 5: Compute the optimal Kalman gain $K_{i}$ at the next time step(next moment) according to Equation (31). 6: Obtain the corrected state vector estimate ${\hat{x}}_{i}$ after the iterative update equation according to Equation (30). 7: Compute the optimized system optimal covariance matrix $P_{i}$ according to Equation (32). 8: Estimate the brightness value ${\hat{I}}^{t+1}$ of the candidate target at the next time step according to Equation (23). 9: Set a gate centered at the predicted target position. 10: **for** j=1 **to** *m* **do** 11: Calculate the matching degree *M* between the candidate target and the predicted target according to Equation (33). 12: **end for** 13: Select the candidate target with the maximum matching degree, and update the trajectory of the *i*-th candidate target. 14: **end for**
